# Ca^2+^-dependent exocytosis of parasperm globular bodies in the strawberry conch gastropod *Conomurex luhuanus*

**DOI:** 10.1371/journal.pone.0360627

**Published:** 2026-10-12

**Authors:** Ryo Yokoya, Kogiku Shiba, Masaya Morita, Kazuo Inaba

**Affiliations:** 1 Shimoda Marine Research Center, University of Tsukuba, Shimoda, Shizuoka, Japan; 2 Tateyama Marine Laboratory, Institute for Marine and Coastal Research, Ochanomizu University, Tateyama, Chiba, Japan; 3 Sesoko Station, Tropical Biosphere Research Center, University of the Ryukyus, Motobu, Okinawa, Japan; University of Tehran, IRAN, ISLAMIC REPUBLIC OF

## Abstract

Several animal groups produce two morphologically distinct types of sperm in the testis, including fertilizing eusperm and non-fertilizing parasperm. This phenomenon, termed sperm dimorphism, is considered to play a role in reproductive strategies such as sperm competition. In the marine gastropod *Conomurex luhuanus*, parasperm possess numerous globular bodies and the cilia-based undulating membranes. However, the function of the globular bodies and the motility of the undulating membranes remain unclear. Here, we describe the process and regulation of globular body exocytosis in parasperm. Upon dilution in artificial seawater, exocytosis occurs via membrane fusion between globular body membrane and the plasma membrane, followed by the release of globular body contents. Exocytosis was markedly suppressed under Ca^2+^ -deficient conditions but was enhanced by increases in intracellular Ca^2+^ induced by an ionophore A23187. In contrast, both the absence of extracellular K⁺ and high extracellular K⁺ concentrations enhanced exocytosis. On the other hand, the direction of wave propagation of the undulating membrane was reversed by high extracellular Ca^2+^ concentrations or by treatment with A23187, whereas it was suppressed under high extracellular K⁺ conditions. Together, these findings indicate that globular body exocytosis and changes in swimming direction are regulated by different mechanisms. This study provides novel insight into the post-ejaculatory behavior and potential function of parasperm within the female reproductive tract of gastropods.

## Introduction

In several groups of animals, two types of sperm, i.e., fertilizable eusperm and non-fertilizable parasperm, are simultaneously produced in the testis. This phenomenon, known as sperm dimorphism, was first reported in the freshwater snail *Paludina vivipara* [1] and has since been documented in a wide range of animals, including insects and mollusks [2]. Although parasperm are thought to play important roles in male reproductive strategy, their behavior and function remain poorly understood at the molecular level.

In a certain species, parasperm act as supportive sperm that facilitate fertilization. In the silkworm *Bombyx mori*, parasperm adhere to numerous eusperm and transport them as carriers toward the seminal receptacle [3]. In the marine snail *Littorina obtusata*, parasperm are transferred to females as nuptial gifts and are believed to promote oocyte maturation by serving as a nutritional resource [2]. In the fruit fly *Drosophila pseudoobscura*, parasperm protect eusperm from the spermicidal activity present in the female reproductive tract [4]. Parasperm may also function to inhibit fertilization by rival male sperm. In the marine sculpin *Hemilepidotus gilberti*, parasperm released into seawater form cellular aggregates on the surface of egg masses that capture eusperm from rival males and inhibit their motility [5]. In the marine snail *Fusitriton oregonensis*, parasperm form a sperm plug within the female reproductive tract, preventing fertilization by eusperm from subsequent males [2]. Collectively, these studies demonstrate the biological significance of parasperm in reproduction.

In eusperm, sperm motility is known to be regulated by transmembrane ionic transport and intracellular signaling pathway [6–10]. For example, membrane hyperpolarization induced by K^+^ channels increases intracellular Ca^2+^ and cAMP level, resulting in the activation of the axonemes in ascidian sperm [9]. An increase in intracellular Ca^2+^ also modulates dynein activity, generating the flagellar asymmetry required for changes in swimming behaviors such as chemotaxis [11,12]. However, the mechanisms underlying globular body loss and the regulation of undulating membrane motility in parasperm remain unknown. In this study, we investigated the physiological mechanism underlying the loss of globular bodies and found that this loss is caused by exocytosis of globular bodies. In addition, we analyzed changes in the motility of the undulating membrane and suggest that globular body loss and changes in undulating membrane motility occur independently.

Parasperm in gastropods particularly show diverse and unique morphologies [2,13–22]. The subclass Caenogastropoda produces parasperm that share common cellular features, including lateral undulating membranes, composed of numerous axonemes and large globular bodies [2,23–25]. Previous studies on parasperm of the marine snail *Conomurex luhuanus* indicated that these cells swim by bending and propagating of undulating membranes [24,26]. Moreover, parasperm reverse the direction of wave propagation, resulting in the backward swimming with a velocity approximately three times faster than forward swimming [26]. This distinctive motility pattern may reflect the behavior of parasperm in the female reproductive tract after ejaculation. Our previous study also showed that globular bodies are formed during parasperm development and that mature parasperm partially lack these structures [26]. However, the biological significance of globular bodies and the motility of undulating membrane in internal fertilization remains largely unexplored.

## Materials and methods

### Solutions

The standard artificial seawater (ASW) contained 462.01 mM NaCl, 9.39 mM KCl, 10.81 mM CaCl_2_, 48.27 mM MgCl_2_ and 10 mM Hepes-NaOH (pH 8.0). ASW with CaCl_2_ or KCl concentrations ranging from 0 to 100 mM was prepared by substituting CaCl_2_ or KCl with NaCl and MgCl_2_ to maintain constant osmolarity. Ca^2+^ -free seawater contained 474.07 mM NaCl, 9.39 mM KCl, 48.27 mM MgCl_2_, 10 mM EGTA, and 10 mM Hepes-NaOH (pH 8.0). The calcium ionophore A23187 (C7522, Sigma-Aldrich, St. Louis, MO, USA) was dissolved in DMSO to prepare a 1 mM stock solution and used at a final concentration of 20 μM in ASW. A T-type voltage-dependent Ca^2+^ channel blocker mibefradil (M5441, Sigma-Aldrich) and NNC55–0396 (N0287, Sigma-Aldrich, St. Louis, MO, USA) was dissolved in distilled water to prepare 2.5 mM and 5 mM stock solutions and used at final concentrations of 5 μM and 10 μM, respectively. A L-type voltage-dependent Ca^2+^ channel blocker nifedipine (N7634, Sigma-Aldrich) was dissolved in DMSO to prepare 2 mM and 20 mM stock solutions and used at final concentrations of 10 and 100 μM, respectively.

### Experimental animals and sperm preparation

The marine gastropods *Conomurex luhuanus* were collected near the Shimoda Marine Research Center (Shimoda, Shizuoka) and near Shizuura Diving Center (Numazu, Shizuoka) from May to September during 2022–2025, and near the Sesoko Station of the University of the Ryukyus (Motobu, Okinawa) from January and March during 2022–2025. Sex was determined based on the presence or absence of a protruding penis on the right side of the head-foot region. Adult males were dissected, and mature sperm were obtained from the vas deferens. During the peak of the reproductive season, up to 20 μl of semen could be collected from each male. The collected sperm were suspended in ASW and were kept on ice.

### Light microscopic observation of globular bodies in parasperm

Diluted sperm suspensions were placed on glass slides and observed under an upright differential interference contrast (DIC) microscope (BX53, Olympus, Tokyo, Japan) using 10 × , 20× and 60 × objective lenses. Images and videos were captured with a digital microscope camera (DP74, Olympus). The recorded videos were analyzed using HAS-Xviewer software (Ditect, Tokyo, Japan).

### Analysis of globular body secretion

Sperm obtained from the sperm duct were diluted 100-fold in ASW with modified Ca^2+^ or K⁺ concentrations, or in ASW containing A23187, mibefradil, NNC55–0396 or nifedipine, and incubated for 15 min at room temperature. The sperm suspensions were then placed on glass slides and recorded at 500 fps using an upright phase-contrast microscope (BX51, Olympus) equipped with a high-speed camera (HAS-U2, Ditect). Recorded videos were analyzed using HAS-Xviewer software, and globular bodies were counted manually using a cell counter.

### Analysis of wave propagation direction in the undulating membrane

Sperm obtained from the sperm duct were diluted 100-fold in ASW with modified Ca^2+^ concentrations or ASW containing A23187. Immediately after dilution, sperm suspensions were placed on glass slides and recorded at 200 fps using an upright DIC microscope (BX51) equipped with a high-speed camera (HAS-U2). Recorded videos were analyzed using HAS-Xviewer software. Wave propagation in the undulating membrane was classified into three categories: forward swimming (wave propagation from the anterior to the posterior end), backward swimming (wave propagation from the posterior to the anterior end), and immotile (no detectable wave propagation).

### Transmission electron microscopy (TEM) of parasperm globular bodies

TEM was performed according to Konno et al. [27], with minor modifications. Sperm obtained from the vas deferens were suspended in ASW. Aliquots of 100 μl of the sperm suspension were allowed to stand for 30 min, followed by the addition of 900 μl of pre-fixative solution (final 2.0% glutaraldehyde in ASW) and fixation at 4 °C for 15 min. Samples were centrifuged at 15,000 rpm for 10 min at 4 °C (KITMAN-18, TOMY Seiko, Tokyo, Japan). The resulting pellet was then fixed with 1 ml of primary fixative containing 2.5% glutaraldehyde, 0.45 M sucrose, and 0.1 M sodium cacodylate-NaOH (pH 7.4) at 4 °C for 2 h. After 30 min of fixation, the pellet was gently detached from the tube using a 27G syringe (NN-2738, Thermo) to ensure sufficient penetration of the fixative. Samples were washed three times at 15 min intervals with washing buffer (0.45 M sucrose, 0.1 M sodium cacodylate-NaOH, pH 7.4), and post-fixed with 1% OsO_4_ in 0.1 M sodium cacodylate-NaOH (pH 7.4) at 4 °C for 1 hr. After dehydration through a graded ethanol series (50–100%), samples were substituted with propylene oxide and embedded in Epon resin (Agar Low Viscosity Resin Kit, Agar Scientific, UK). Polymerization was performed at 60 °C for 24 hr. Ultrathin sections (approximately 70 nm) were prepared using an ultramicrotome (ULTRACUT-S, Leica Microsystems), mounted on copper grids (EM Fine Grid, Nisshin EM, Tokyo, Japan), stained with 4% aqueous uranyl acetate and Reynolds’ lead citrate, and observed under a transmission electron microscope (JEM-2100, JEOL, Tokyo, Japan).

### Statistical analysis

Individual animals were treated as biological replicates, whereas multiple sperm cells analyzed from the same individual were treated as repeated observations. To account for the non-independence of observations obtained from the same individual, individual identity was included as a random effect in all generalized linear mixed models (GLMMs).

For analyses of globular body filling, the numbers of globular bodies retained and released were analyzed as grouped binomial data using cbind(retained, released). Initial analyses using binomial GLMMs showed substantial overdispersion; therefore, beta-binomial GLMMs with a logit link were used. Model adequacy and residual dispersion were evaluated using simulation-based residual diagnostics implemented in the DHARMa package.

For the extracellular Ca^2+^ experiment, residual variability differed among CaCl_2_ treatments even after fitting a beta-binomial GLMM with a common dispersion parameter. Therefore, a dispersion submodel was included to allow the beta-binomial dispersion parameter to vary among CaCl_2_ treatments. This model adequately accounted for both overall overdispersion and treatment-dependent heterogeneity in residual variance.

Estimated marginal means were calculated on the response scale and are presented as model-estimated proportions with 95% confidence intervals (CIs). Comparisons between each experimental treatment and the designated control were performed using Dunnett-adjusted multiple comparisons. Statistical significance was defined as P < 0.05, P < 0.01, and P < 0.001. All statistical analyses were performed in R using the glmmTMB, emmeans, and DHARMa packages. Graph preparation and statistical analyses were conducted using R software (https://cran.r-project.org).

The underlying data for all figures are provided in a multi-sheet Excel file in the Supporting Information (S1 Data).

## Results

### Gradual loss of the globular bodies in *C. luhuanus*

Both eusperm and parasperm collected from vas deferens show changes in the morphology and motility after dilution into ASW (Fig 1A–C). Eusperm are released as bundles (Fig 1A), which dissociate within 5 min after dilution in ASW (Fig 1B). The direction of movement and flagellar symmetry also change over the following 2 h [26,28]. In parasperm, numerous globular bodies, typically up to 70 (50.3 ± 6.8, means ± standard deviation) per cell, were observed in the cytoplasm. Changes in movement direction and velocity, as well as loss of globular bodies, were induced after dilution in ASW (Fig 1C). Following the loss of globular bodies, some parasperm adhered to each other and formed aggregates (Fig 1D). Eusperm also aggregated in the vicinity of parasperm (Fig 1E).

**Fig 1:**
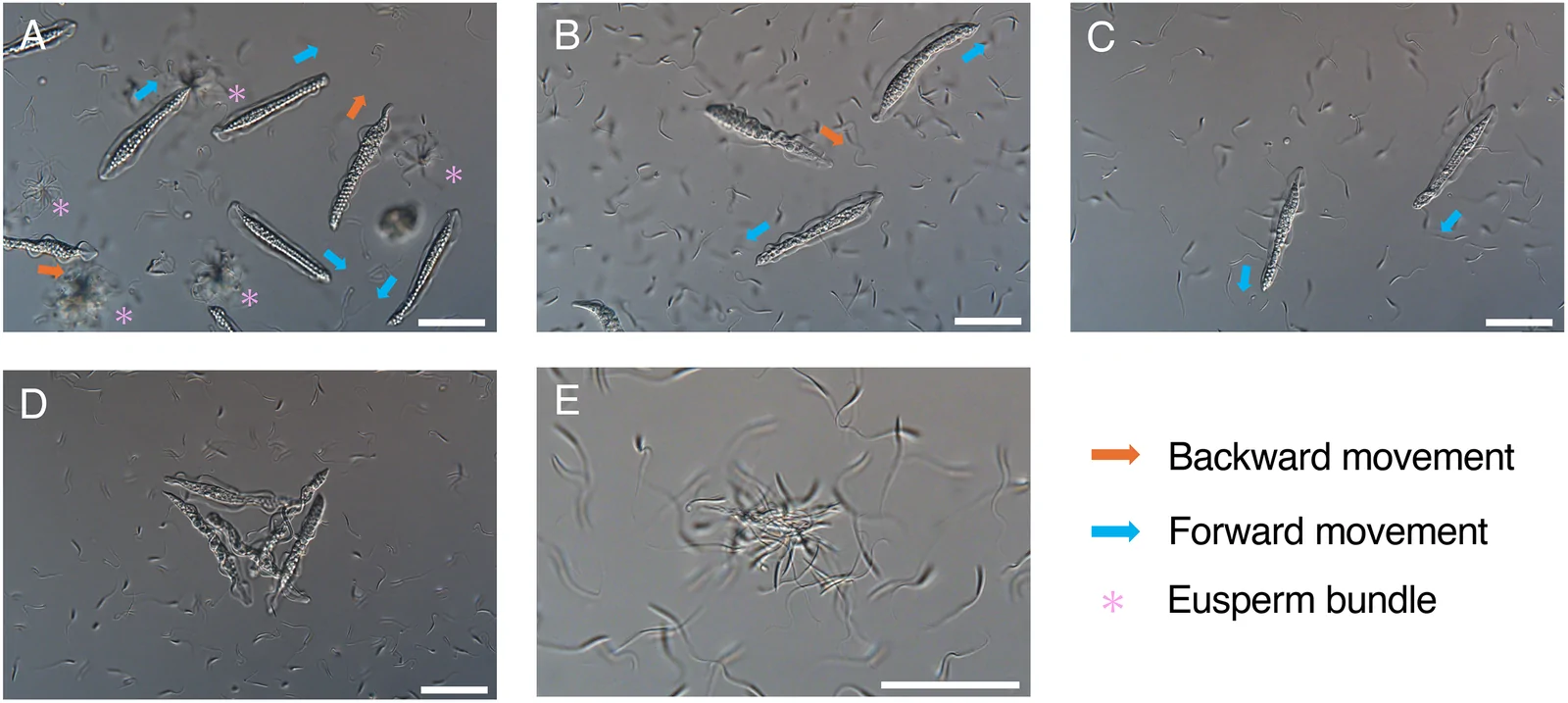
Changes in behavior of *Conomurex luhuanus* sperm after dilution into ASW. (A) Immediately after dilution in artificial seawater (ASW), bundles of eusperm and the parasperm with both forward and backward movement were observed. (B) Approximately 10 min after dilution, eusperm bundles were dissociated, and the granular bodies in parasperm started exocytosis. (C) Approximately 60 min after dilution, most parasperm showed forward swimming. (D, E) Approximately 120 min after dilution, some parasperm showed adhesion to each other (D), and the eusperm partially formed aggregates different from initially observed sperm bundles (E). Images obtained by differential interference contrast microscopy. Blue and orange arrows indicate backward and forward movements, respectively. Asterisks, eusperm bundles. Scale bars, 50 μm.

To investigate the process of globular body loss, we examined the globular body filling rate at various time points after dilution in ASW (Fig 2A; also see Materials and methods). The globular bodies appear glossy, spherical bodies under different interference contrast microscopy (Fig 2A, left) [26]. The loss of globular bodies, easily recognized by the appearance of dark circular regions (Fig 2A, middle and right), occurred at multiple sites within individual parasperm but tended to begin in the posterior region. To quantify this process, the globular body filling rate determined for each parasperm by counting the number of retained and lost globular bodies. The filling rate was calculated as the ratio of retained globular bodies to the total number of retained and lost globular bodies.

**Fig 2:**
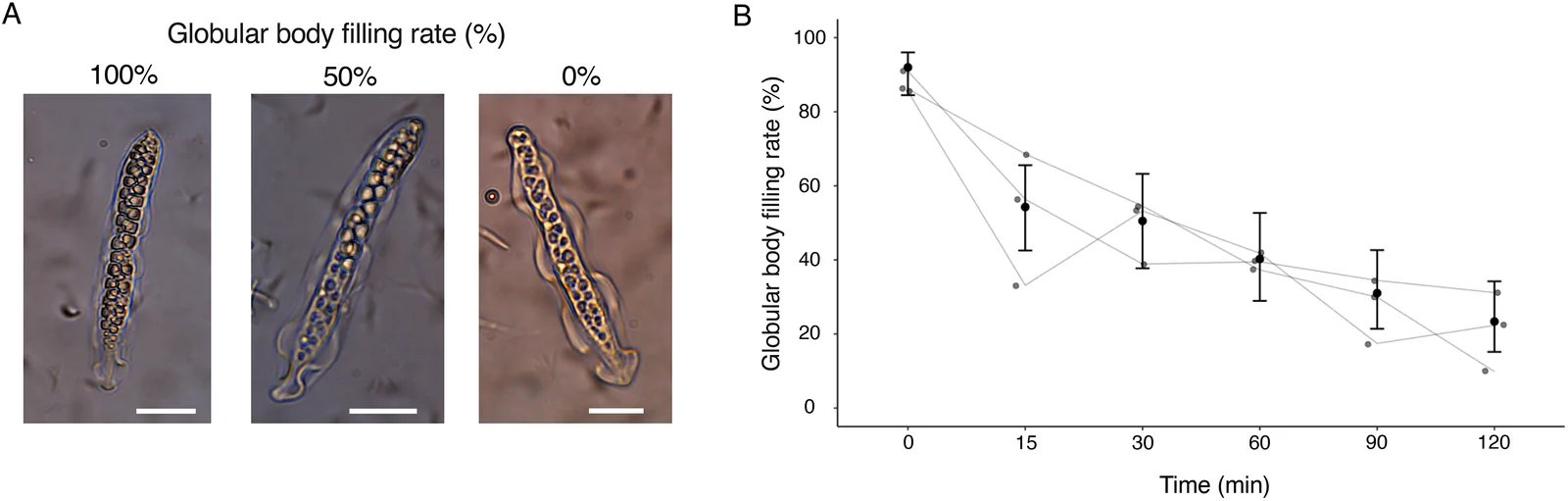
The filling rate of globular bodies gradually decreases over time. (A) The number of globular bodies retained were counted for each parasperm. The filling rate of globular bodies (%) = Number of globular bodies containing contents / Total number of globular bodies × 100. Scale bar, 20 μm. (B) The plot shows a gradual decrease in the globular body filling rate over time after dilution into artificial seawater. Black circles and vertical error bars represent the overall means and 95% confidence intervals (CIs) across experiments. Gray circles indicate the mean values for individual experiments, with lines connecting values from the same experiment across treatments. The estimated marginal mean values predicted from GLMM analysis are 92.0% [95% CI, 84.5–96.0] (0 min), 54.3% [95% CI, 42.5–65.6] (15 min), 50.5% [95% CI, 37.7–63.2] (30 min), 40.2% [95% CI, 28.9–52.7] (60 min), 31.0% [95% CI, 21.4–42.6] (90 min), 23.4% [95% CI, 15.2–34.2] (120 min). The numbers of analyzed sperm are N = 30 from three independent experiments. The individual numerical values underlying the figure are provided in S1 Data (Fig 2B).

At 0 minutes post-dilution, the average globular body filling rate was over 92.0% [95% CI, 84.5–96.0], indicating that globular bodies were largely retained. After 15 minutes, the average globular body filling rate decreased to 54.3% [95% CI, 42.5–65.6]. After 120 minutes, the average filling rate was 23.4% [95% CI, 15.2–34.2], indicating that the loss of globular body does not occur abruptly after dilution in ASW nor at a specific time point, but rather progresses gradually over time (Fig 2B).

### Exocytotic release of globular body contents

In this study, we observed the process of globular body loss in detail using a phase-contrast microscope equipped with a high-speed camera. Shortly after dilution in ASW, some globular bodies, initially glossy and spherical, rapidly lost their luster and appeared dark (Fig 3A, B). This phenomenon was observed more frequently 30 min after dilution in ASW. High-speed imaging revealed that the bright contour of the globular body disappeared, followed by the gradual diffusion of its dark internal contents (Fig 3C). The disappearance of individual globular bodies occurred rapidly, within 0.2 s (Fig 3C; S1 File).

**Fig 3:**
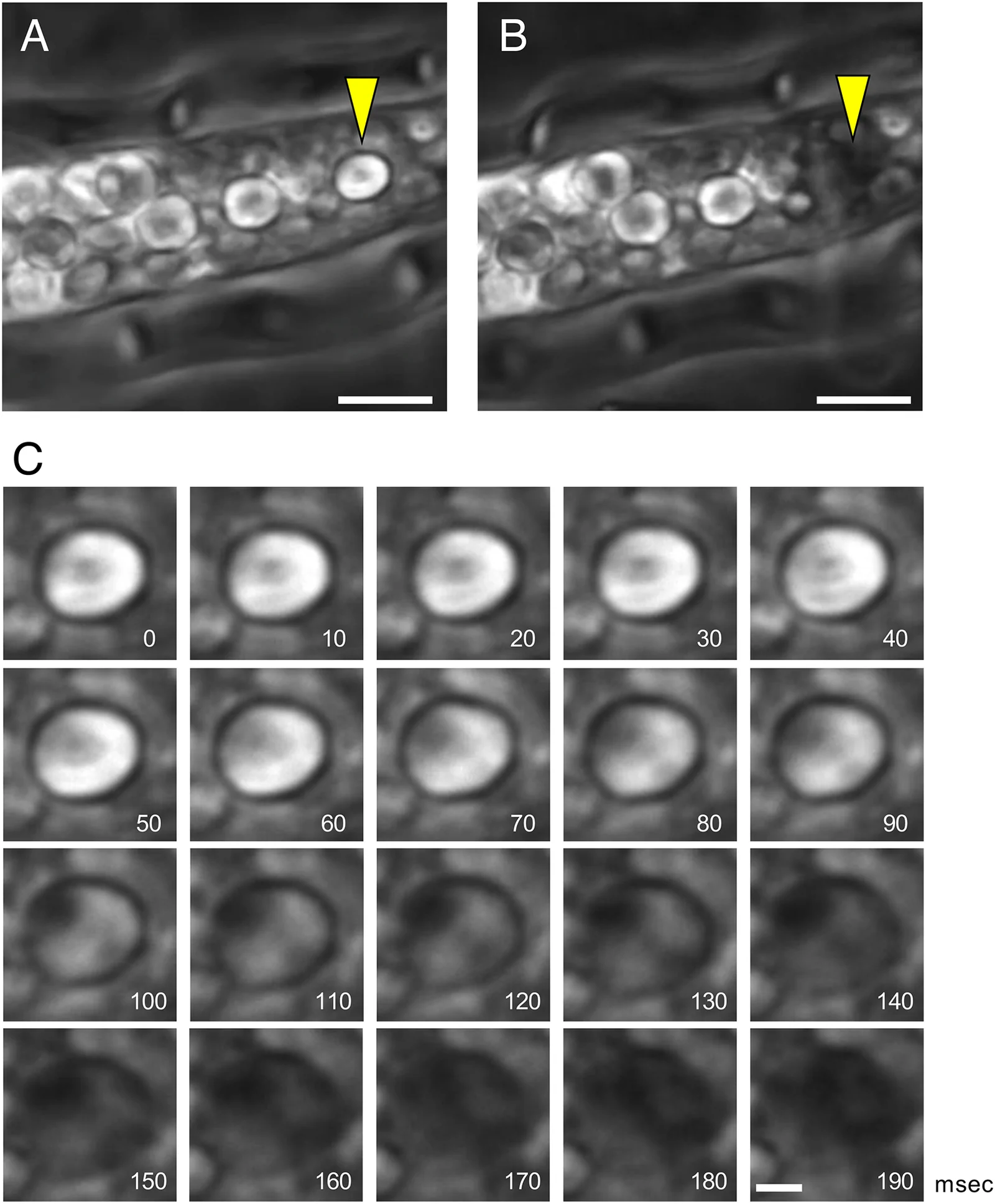
Phase-contrast microscopic images for disappearance of globular bodies. (A, B) Images of a globular body (yellow arrowhead) before (A) and after (B) exocytosis. Scale bar, 20 μm. (C) Sequential images of the secretion of globular bodies recorded by a high-speed camera at 500 fps. Images with 10 ms intervals are shown. Also see S1 File. Scale bar, 5 μm.

TEM observation of the parasperm showed two lateral undulating membranes, each containing up to ~150 axonemes, along with globular bodies whose vesicular membranes are located close to the plasma membrane [26] (Fig 4A). After dilution in ASW, parasperm exhibited the disappearance of some globular bodies (Fig 2B). TEM analysis of parasperm at 30 min post-dilution showed that the vesicular membranes of several globular bodies fused with the plasma membrane, forming a fusion pore and leaving the fragmented plasma membrane structures (Fig 4B). Subsequently, the globular bodies underwent complete fusion, exposing the contents of globular bodies containing electron-dense materials (Fig 4C). The electron-dense granules did not appear to disperse rapidly but instead remained associated as small masses. Some granules remained attached to the inner surface of proximal vesicular membrane, which became a newly exposed plasma membrane (Fig 4C).

**Fig 4:**
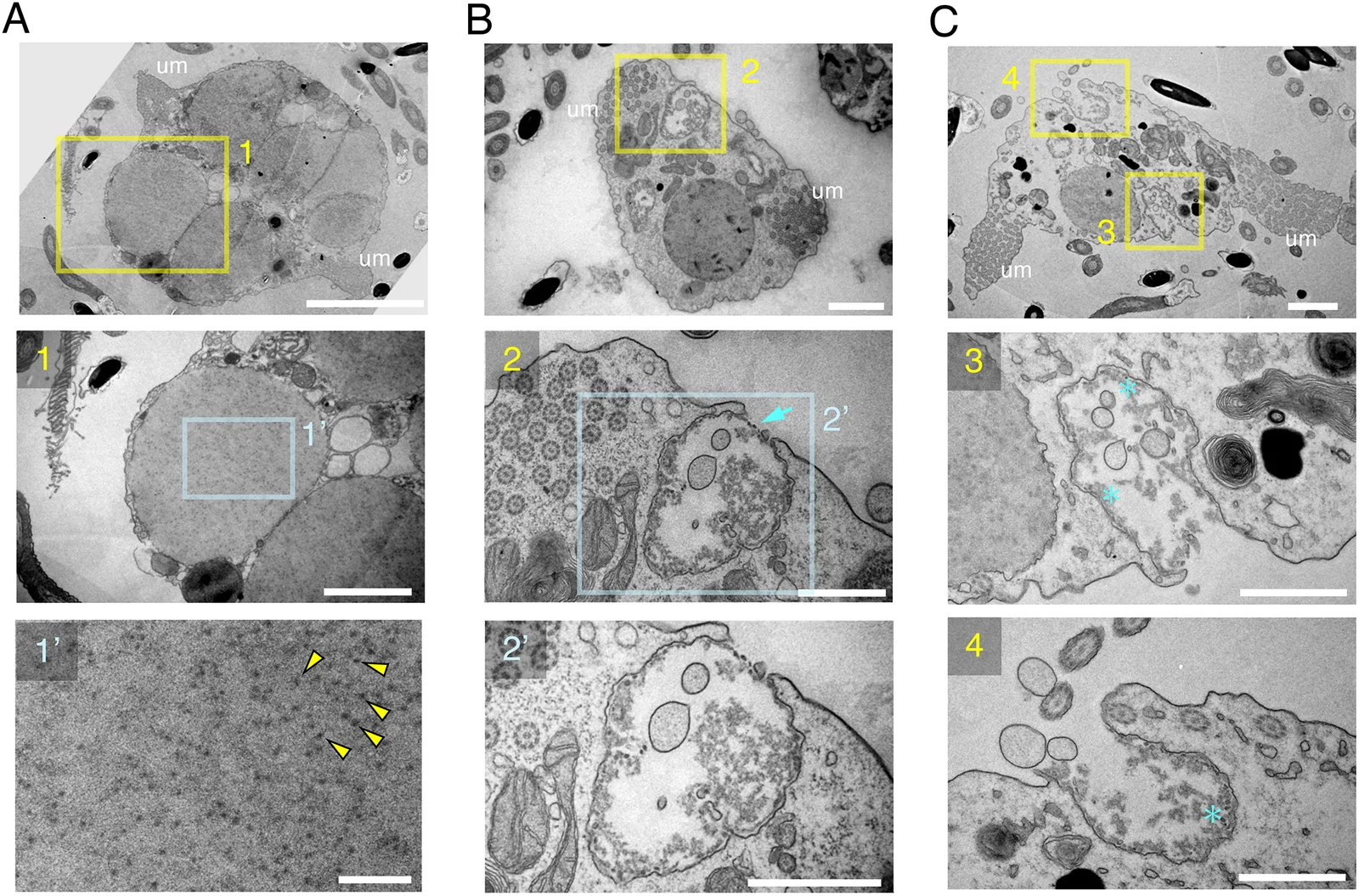
Transmission electron microscopic observation of the secretion of globular bodies. (A) Parasperm before globular body exocytosis. Left, cross-section of a parasperm. Middle, magnified image of a globular body labeled 1 in the left image. Right, magnified image of a globular body content labeled 1’ in the middle image. Scale bar, 5.0 μm (left); 2.0 μm (middle); 0.5 μm (right). (B) Parasperm at initial stage of membrane fusion with a fusion pore (arrow). Left, cross-section of a parasperm. Middle, magnified image of a globular body labeled 2 in the left image. Right, magnified image of a globular body content labeled 2’ in the middle image. Scale bar, 2.0 μm (left and middle); 0.5 μm (right). (C) Parasperm after complete fusion of globular body. Left, cross-section of a parasperm. Middle, magnified image of a globular body opening labeled 3 in the left image. Right, magnified image of a globular opening labeled 4 in the left image. Scale bar, 2.0 μm (left); 1.0 μm (middle and right). um, undulating membrane. Yellow arrowheads, electron-dense granules in a globular body; blue arrow, globular body opening with a fusion pore; asterisks, electron-dense granules retained in association with membrane. Images of a total of 43 cells were obtained. Of these, 23 cells were examined and documented in detail. Representative cells from high-quality preparations showing typical morphology are presented in the figures.

### Changes in K^+^ concentration accelerate exocytosis of globular body

It is well established that K^+^ -dependent membrane potential plays an important role in diverse aspects of sperm intracellular signaling [6–10] We therefore investigated the effect of K⁺ concentration in ASW on the secretion of globular body contents in parasperm. At 15 min after dilution in standard ASW, the globular body filling rate was 55.6% [95% CI, 43.4–67.1]. This value showed no significant change in ASW containing 20 mM K⁺ (Fig 5). In contrast, increasing the KCl concentration to 40 mM and 100 mM markedly decreased the filling rate to 21.4% [95% CI, 14.0–31.4] and 7.2% [95% CI, 3.98–12.6], respectively. Intriguingly, the filling rate was also significantly reduced to 31.1% [95% CI, 21.4–42.7] in K ⁺ -free ASW (Fig 5). These results indicate that both increase and decrease in extracellular K⁺ concentration promote the secretion of globular body contents, likely through alteration of membrane potential.

**Fig 5:**
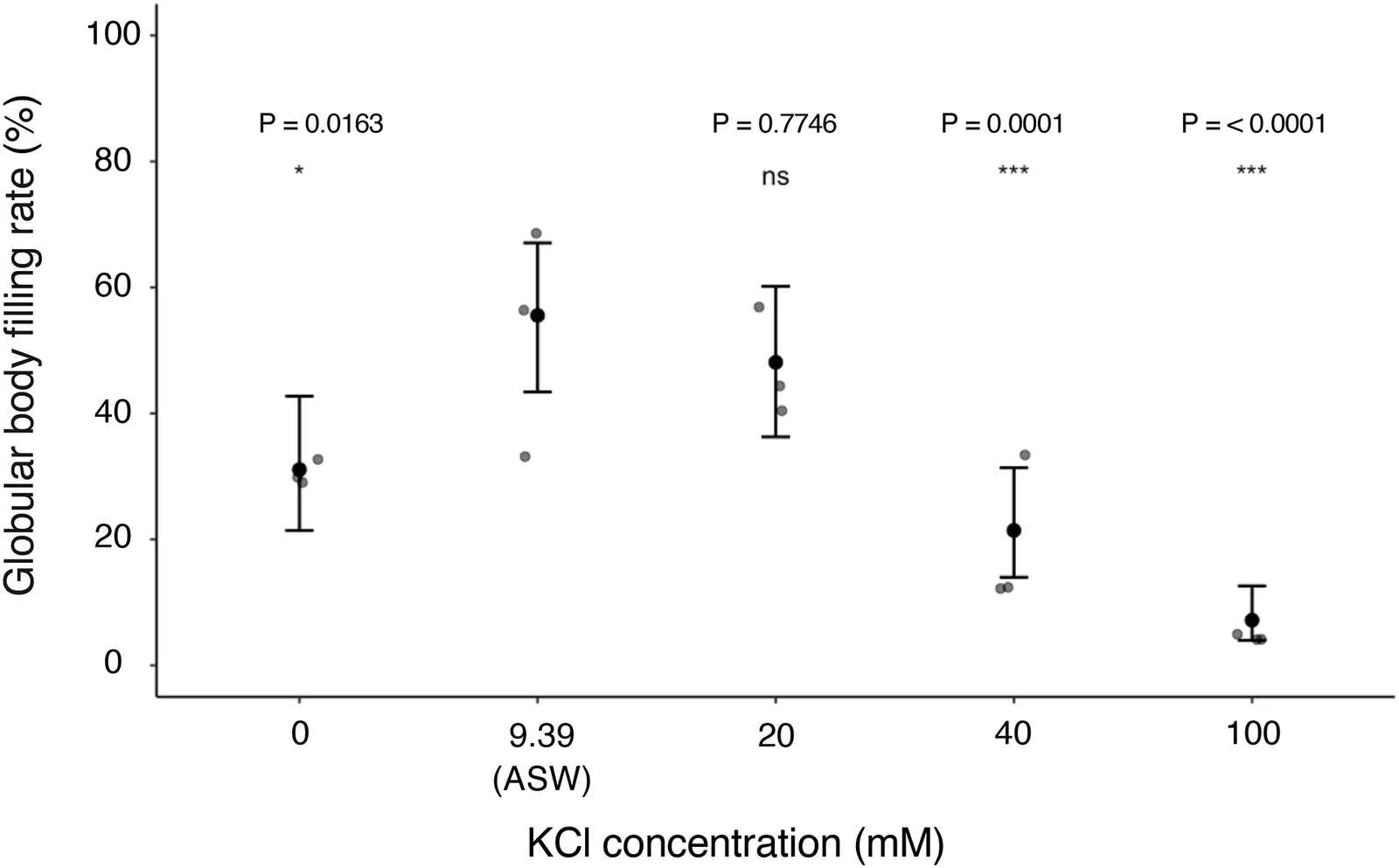
Effect of extracellular K^+^ concentration on the exocytosis of globular bodies. Globular body filling rate was measured 15 min after dilution in artificial seawater (ASW) containing various KCl concentrations. Black circles and vertical error bars represent the overall means and 95% confidence intervals (CIs) across experiments. Gray circles indicate the mean values for individual experiments. The beta-binomial generalized linear mixed model (GLMM) analysis revealed a significant effect of treatment. The mean values predicted from GLMM analysis are 31.1% [95% CI, 21.4–42.7] (0 mM), 55.6% [95% CI, 43.4–67.1] (ASW), 48.1% [95% CI, 36.3–60.2] (20 mM), 21.4% [95% CI, 14.0–31.4] (40 mM), and 7.2% [95% CI, 3.98–12.6] (100 mM). *** p < 0.001, * p < 0.01, * p < 0.05 compared with 9.39 mM KCl (ASW, control) using Dunnett’s test. Exact p-values are indicated above. ns, not significant. The numbers of analyzed sperm are N = 30 from three independent experiments. The individual numerical values underlying the figure are provided in S1 Data (Fig 5).

### Ca^2+^ influx is required for globular body exocytosis

To examine the requirement of Ca^2+^ for the induction of globular body secretion, we analyzed the effects of ASW containing various concentrations of CaCl_2_ (Fig 6). In Ca^2+^ -free ASW, little or no globular body exocytosis was observed after 15 min of incubation. In contrast, increasing the CaCl_2_ concentration to 20 mM, 40 mM and 100 mM did not enhance exocytosis beyond the level observed in standard ASW (Fig 6). Addition of A23187 to ASW significantly induced the exocytosis, suggesting that Ca^2+^ influx is required for globular body exocytosis. These results suggest that Ca^2+^ is necessary for the induction of globular body secretion.

**Fig 6:**
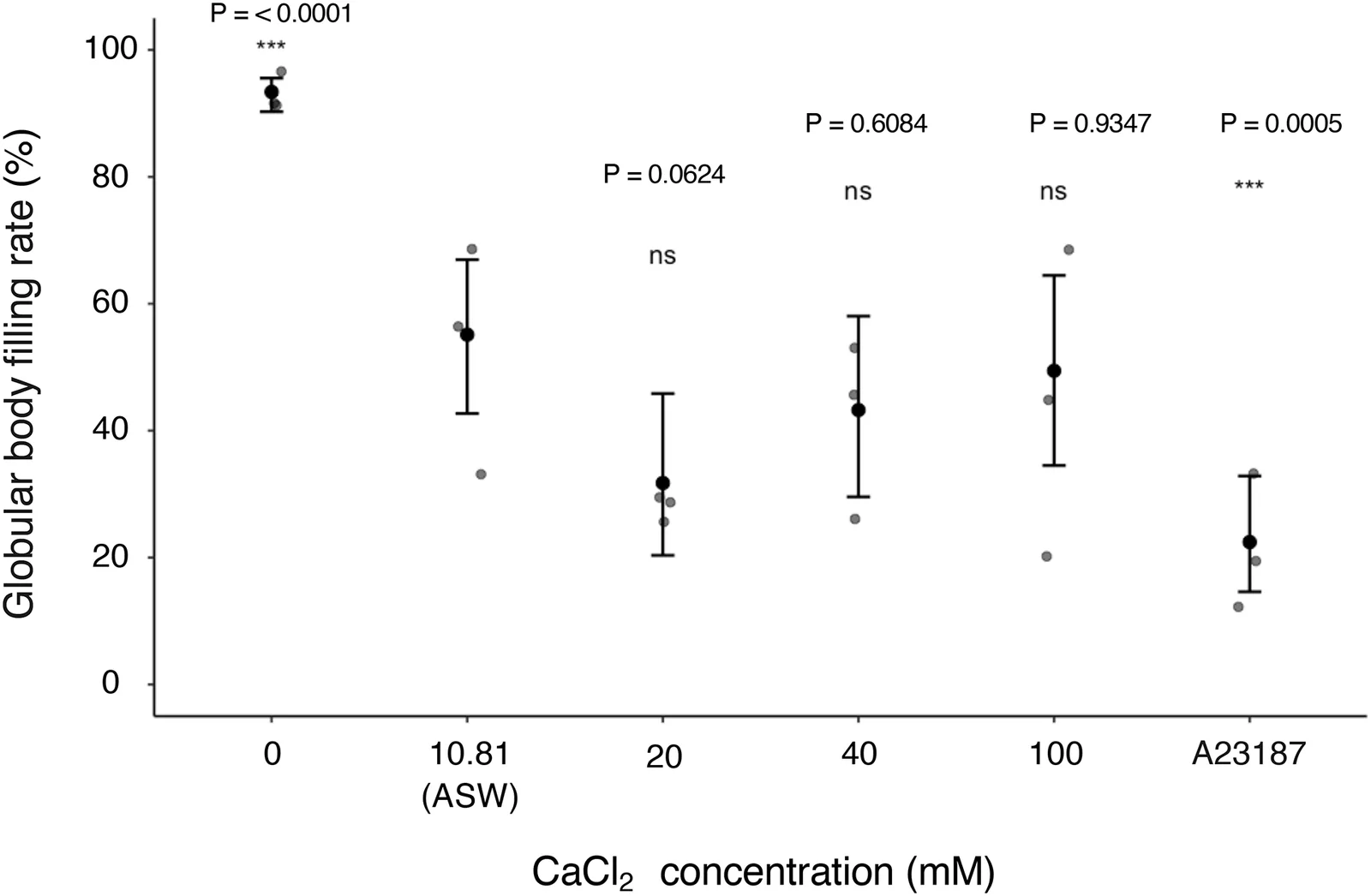
Effect of extracellular Ca^2+^ concentration on the exocytosis of globular bodies. Globular body filling rate was measured 15 min after dilution in artificial seawater (ASW) containing various CaCl_2_ concentrations, as well as in artificial seawater containing 20 μM A23187. Black circles and vertical error bars represent model-estimated proportions and 95% confidence intervals (CIs) across experiments. Gray circles indicate the mean values for individual experiments. The beta-binomial generalized linear mixed model (GLMM) analysis revealed a significant effect of treatment. The mean values predicted from GLMM analysis are 93.8% [95% CI, 90.3–95.5] (0 mM), 55.1% [95% CI, 42.7–66.9] (ASW), 31.7% [95% CI, 20.4–45.9] (20 mM), 43.2% [95% CI, 29.6–58.0] (40 mM), and 49.4% [95% CI,34.5–64.5] (100 mM). for solution with several Ca^2+^ concentrations, and 22.4% [95% CI, 14.6–32.9] for 10.81 mM CaCl_2_ in the presence of 20 μM A23187. *** p < 0.001, ** p < 0.01, * p < 0.05 compared with 10.81 mM CaCl_2_ (ASW, control) using Dunnett’s test. Exact p-values are indicated above. ns, not significant. The numbers of analyzed sperm are N = 30 from three independent experiments. The individual numerical values underlying the figure are provided in S1 Data (Fig 6).

Based on the observed effects of Ca^2+^ and K⁺ concentration in ASW, we hypothesized that voltage-dependent Ca^2+^ channels are involved in regulating globular body exocytosis. To test this hypothesis, we examined the effects of two T-type voltage-dependent Ca^2+^ channel inhibitors, mibefradil and NNC55–0396, and a L-type voltage-dependent Ca^2+^ channel inhibitor, nifedipine. The globular body filling rate at 15 min after dilution in ASW increased to 78.4% [95% CI, 69.9–85.0] or 90.0% [95% CI, 84.3–93.8] in the presence of 10 or 50 μM mibefradil, respectively. NNC55–0396 also showed high globular body filling rate; 81.3% [95% CI, 73.1–87.4] or 86.2% [95% CI, 79.3–91.1] at 5 or 10 μM, respectively (Fig 7). In contrast, nifedipine at 10 or 100 μM had no significant effect on the globular body filling rate (Fig 7). These pharmacological results are consistent with the possible involvement of T-type voltage-dependent Ca^2+^ channels in the secretion of globular body contents.

**Fig 7:**
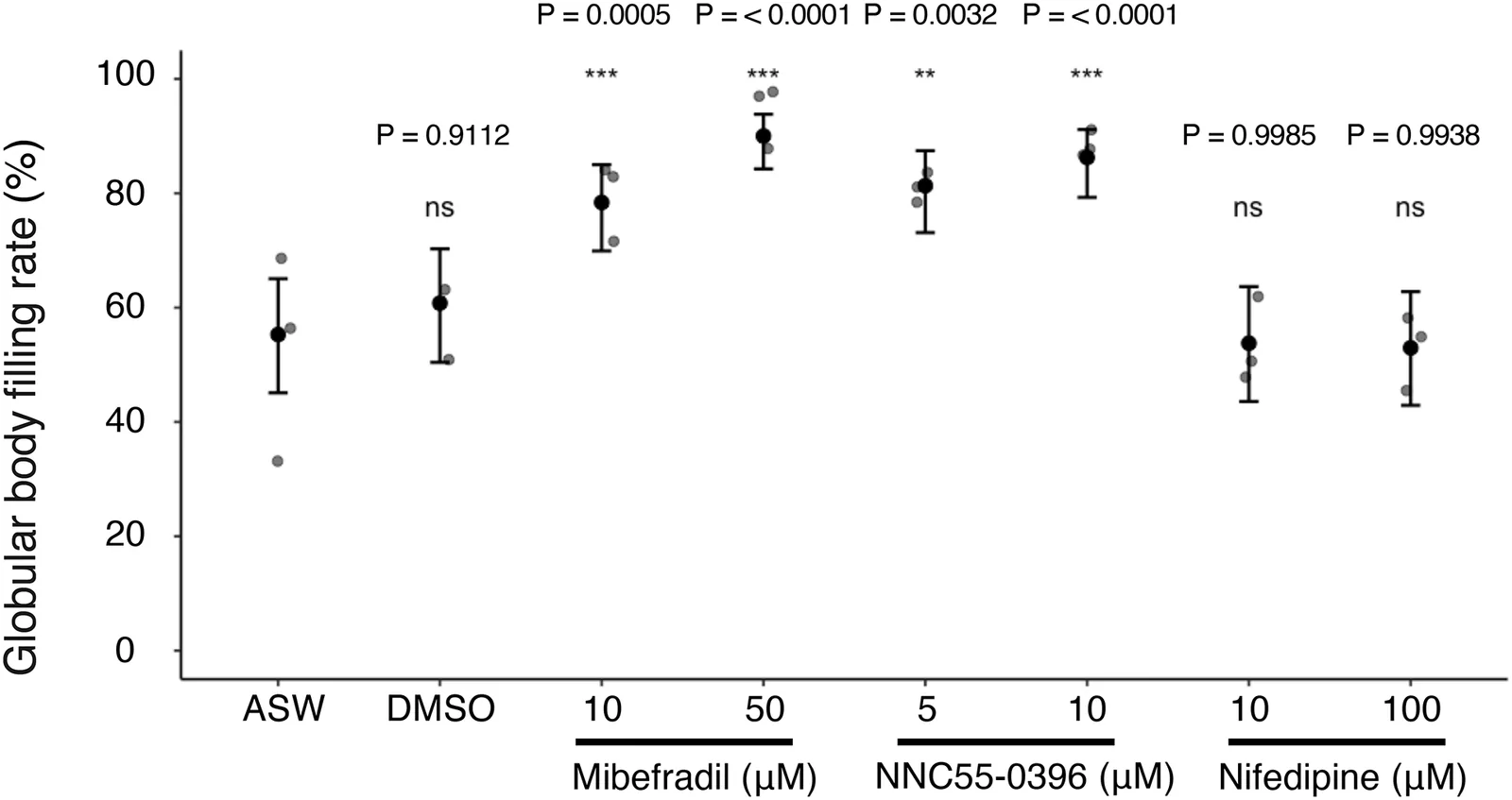
Effect of voltage-gated Ca^2+^ channel inhibitors on the exocytosis of globular bodies. Globular body filling rate was measured 15 min after dilution in artificial seawater containing 0.5% DMSO, a T-type Ca^2+^ channel inhibitor mibefradil and NNC55–0396, and a L-type Ca^2+^ channel inhibitor nifedipine. Black circles and vertical error bars represent model-estimated proportions and 95% confidence intervals (CIs), respectively, from the beta-binomial generalized linear mixed model (GLMM). Gray circles indicate the mean values for individual animals. The beta-binomial GLMM analysis revealed a significant effect of treatment. The mean values predicted from GLMM analysis are 55.3% [95% CI, 45.1–65.1] (ASW), 60.8% [95% CI, 50.4–70.3] (DMSO), 78.4% [95% CI, 69.9–85.0] (10 μM mibefradil), 90.0% [95% CI, 84.3–93.8] (50 μM mibefradil), 81.3% [95% CI, 73.1–87.4] (5 μM NNC55–0396), 86.2% [95% CI, 79.3–91.1] (10 μM NNC55–0396), 53.8% [95% CI, 43.5–63.7] (10 μM nifedipine), and 53.0% [95% CI, 42.9–62.8] (100 μM nifedipine). *** p < 0.001, ** p < 0.01, and * p < 0.05 compared with ASW (control) using Dunnett’s test. Exact p-values are indicated above. ns, not significant. A total of 30 sperm were analyzed per condition from three independent animals. The individual numerical values underlying the figure are provided in S1 Data (Fig 7).

### Difference in K^+^ and Ca^2+^ requirement between globular body exocytosis and swimming direction regulations

Parasperm initially show backward swimming, characterized by high-speed propagation of the undulating membrane from the posterior to the anterior end (S2 File). This rapid swimming is most frequently observed shortly after dilution in ASW, and parasperm subsequently switch to forward swimming [26].

To determine whether K^+^ and Ca^2+^ regulate globular body exocytosis and swimming direction through the same signaling pathways in parasperm, we examined the effects of extracellular K^+^ and Ca^2+^ concentrations, as well as voltage-dependent Ca^2+^ channel inhibitors, on the propagation direction of undulating membrane. In ASW containing 100 mM KCl, parasperm exhibited highly active motility with a strong predominance of forward swimming, characterized by propagation of the undulating membrane from the anterior to the posterior end, suggesting that forward movement is induced by elevated K⁺ concentration (Fig 8, S1 Table). In contrast, a higher proportion of backward swimming was observed in ASW containing 100 mM Ca^2+^ compared with standard ASW. In the presence of 20 μM A23187 in ASW, most parasperm reversed their swimming direction and exhibited backward movement. These results suggest that backward swimming is induced by Ca²^2+^ influx into the cell. Furthermore, mibefradil and NNC55–0396 significantly suppressed motility in both directions, whereas nifedipine had no detectable effect on the swimming direction of parasperm (Fig 8, S1 Table).

**Fig 8:**
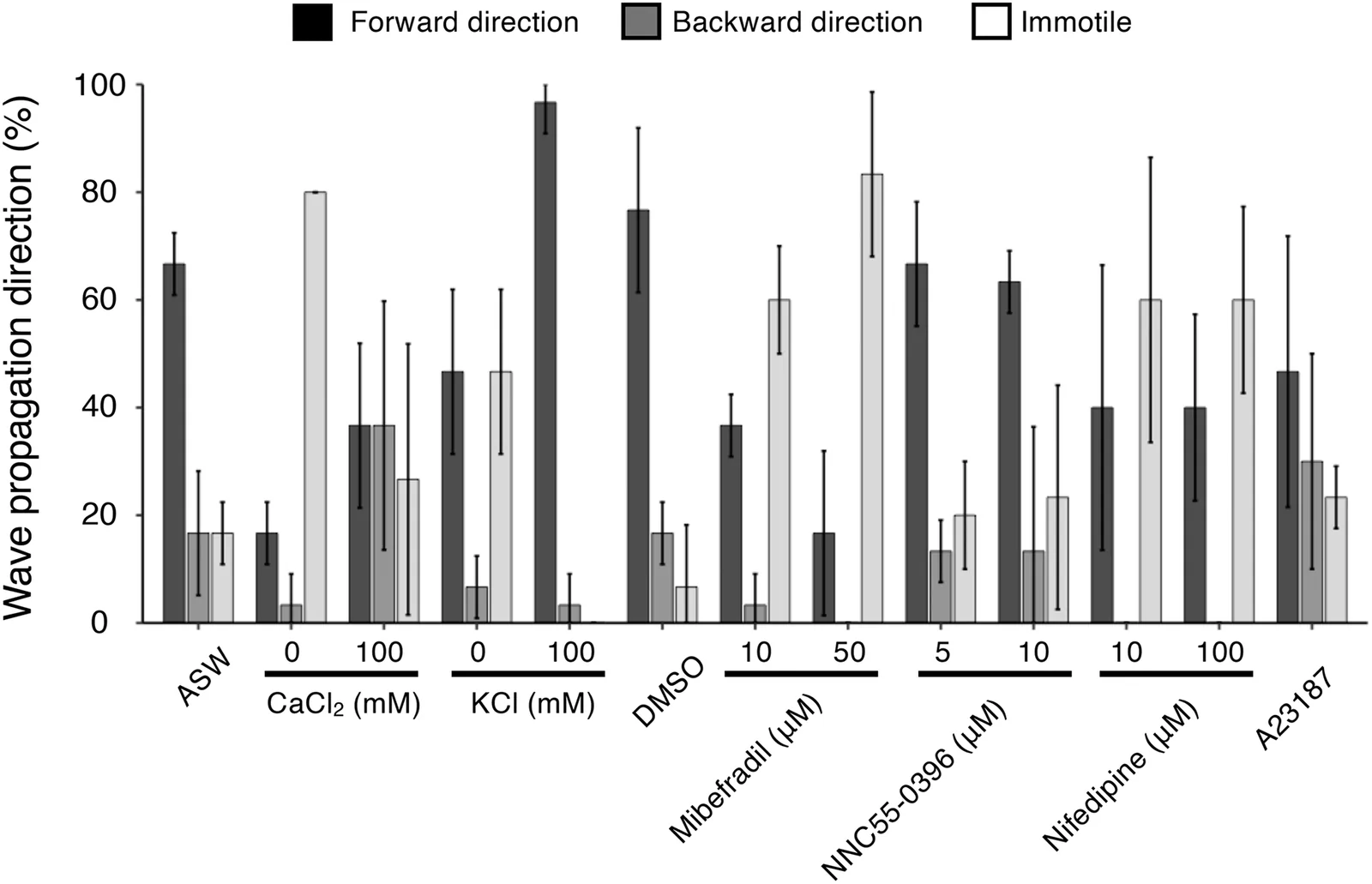
Effects of Ca^2+^ and K^+^ on the direction of wave propagation in the undulating membrane of parasperm. The direction of wave propagation in the undulating membrane was quantified in artificial seawater containing 0 or 100 mM K^+^ or Ca^2+^ , as well as in artificial seawater containing 0.5% DMSO, 20 μM A23187, 10 or 50 μM mibefradil, 5 or 10 μM NNC55–0396, or 10 or 100 μM nifedipine. A total of 30 sperm were analyzed per condition from three independent animals. The values of mean ± SD are shown in S1 Table. The individual numerical values underlying the figure are provided in S1 Data (Fig 8).

## Discussion

In this study, we describe the process and underlying mechanisms of globular body secretion, a dynamic cellular event observed in parasperm of the gastropod *C. luhuanus*. Exocytosis occurred in ASW containing an appropriate concentration of Ca^2+^ . It was not apparently enhanced in a high-Ca² ⁺ ASW and was rather suppressed in a Ca^2+^ -free ASW. However, it was accelerated in ASW supplemented with A23187. In contrast, exocytosis was enhanced under both K^+^ -free and high-K^+^ conditions. Reversal of undulating membrane wave propagation, accompanied by a change in swimming direction, was promoted by high Ca^2+^ and A23187, but suppressed under K^+^ -free or high-K^+^ conditions. These distinct K^+^ and Ca^2+^ dependencies of globular body exocytosis and motility suggest that these two processes are regulated by separate signaling pathways rather than a shared mechanism (summarized in Fig 9A). The differences in regulatory modes between globular bodies and the undulating membrane may reflect the timing of these processes within the female reproductive tract.

**Fig 9:**
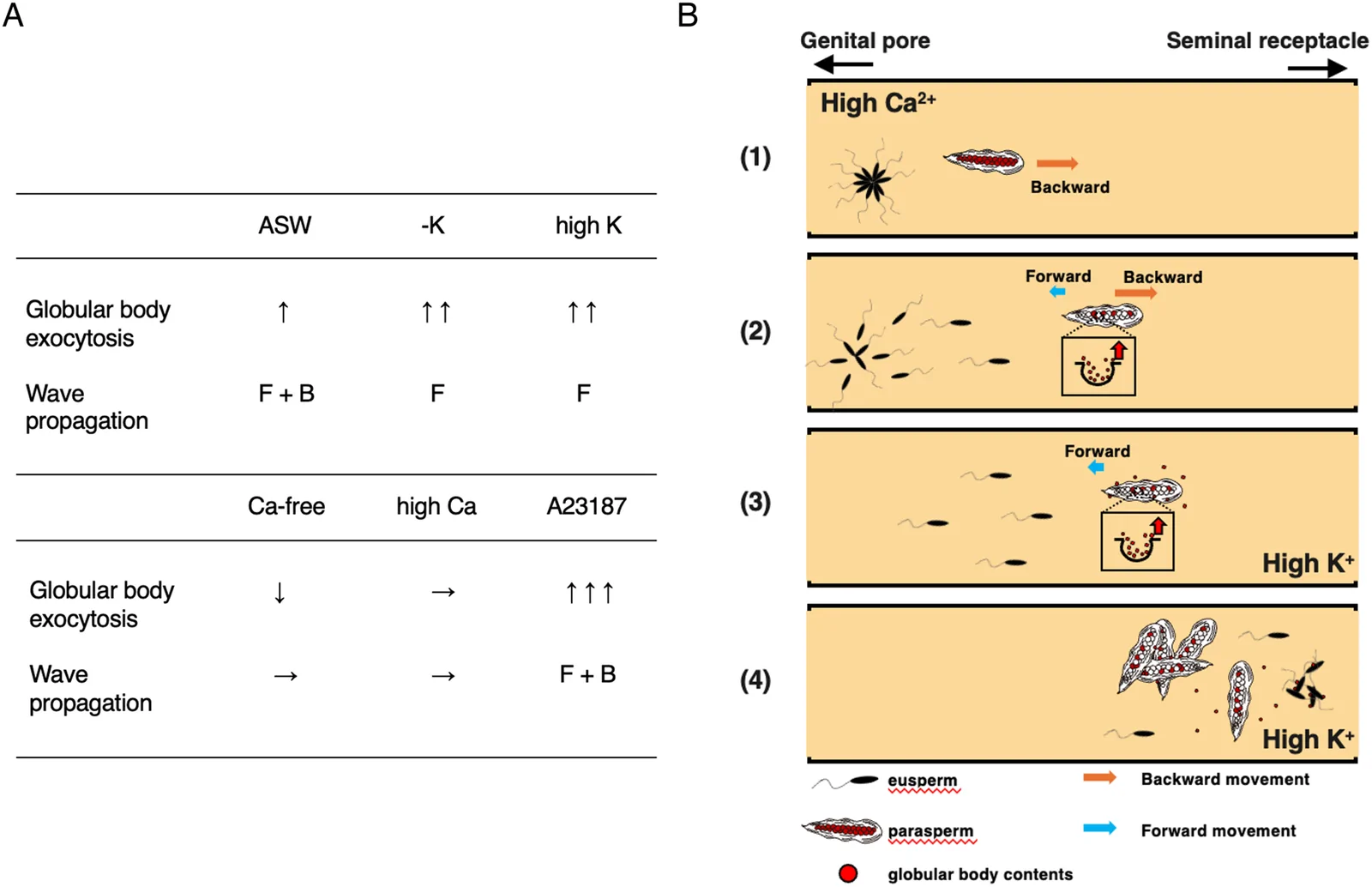
Possible model for post-ejaculatory behaviors of eusperm and parasperm in *C. luhuanus.* A. Summary of K^+^- and Ca^2+^-dependencies of globular body exocytosis and parasperm motility. F and R represent forward (anterior-to-posterior) or backward (posterior-to-anterior) propagation of the undulating membrane, respectively. Upward or downward arrows represent acceleration and suppression, respectively, with the number of arrows indicating the extent of the change. A horizontal arrow indicates no significant change. B. Possible changes in parasperm after ejaculation. (1) Following copulation, both types of sperm are ejaculated into the female genital tract. Eusperm show no progressive motility due to bundle formation. In the proximal area of the genital tract, high concentration of Ca^2+^ is thought to induce dissociation of eusperm bundles. In this condition, parasperm show fast backward swimming ahead of eusperm. (2) Eusperm are dissociated from the bundles and gradually start to swim forward. Parasperm increase the population showing slow forward swimming and the globular bodies gradually undergo exocytosis. (3) Eusperm show progressive forward swimming, whereas most of parasperm stop fast backward swimming. Exocytosis of parasperm globular bodies is accelerated, likely due to the increase of K^+^ concentration. (4) After a certain period of time, certain eusperm and parasperm form their own respective aggregates. These aggregations are considered to occur within inner part of female reproductive tract, including the seminal receptacle, but it has not been elucidated in this study whether these changes are caused by globular body contents.

The regulation of motility in eusperm has been described previously [28]. Shortly after dilution, Ca^2+^ -dependent dissociation of eusperm bundles occurs, although eusperm still lack progressive motility. Thus, sperm bundles and early-stage eusperm may function to retard movement within the female reproductive tract (Fig 9B, panels 1 and 2). In contrast, parasperm exhibit backward motility immediately after dilution in ASW, with a higher velocity than subsequent forward movement [26]. Because A23187-induced Ca^2+^ influx elicits backward motility, this process appears to be Ca^2+^ dependent. Inhibition of T-type Ca^2+^ channels markedly reduces motility and abolishes backward movement, suggesting that early Ca^2+^ influx through these channels triggers backward swimming in parasperm. This difference in swimming behavior between eusperm and parasperm likely enables rapid initial migration of parasperm, preceding eusperm within the female reproductive tract (Fig 9B, panels 1 and 2).

Notably, exocytosis does not occur at this early stage despite elevated Ca^2+^ levels, implying that additional factors, potentially including K^+^ -dependent changes in membrane potential, are required during later stages after ejaculation. Elevated K^+^ promotes forward, slower swimming while enhancing globular body exocytosis, potentially allowing parasperm to advance ahead of eusperm and release globular body contents in inner regions of the tract, such as the seminal receptacle (Fig 9B, panels 3 and 4). Taken together, parasperm are hypothesized to migrate rapidly through the female reproductive tract ahead of eusperm and subsequently undergo exocytosis after reaching specific regions, possibly in response to membrane potential changes associated with increased K^+^ concentration. Supporting this idea, high K^+^ concentrations have been reported in the female reproductive tract of mammals and mollusks [29,30].

Pharmacological analyses suggest that exocytosis is triggered by Ca^2+^ influx through T-type voltage-dependent Ca^2+^ channels. However, elevated extracellular Ca^2+^ alone does not induce exocytosis, indicating that Ca^2+^ is necessary but not sufficient. Exocytosis is a multistep process involving membrane tethering, docking, and fusion; although Ca^2+^ commonly acts as a trigger, additional signaling pathways are likely required [31,32]. Signaling pathways such as phosphatidylinositol signaling, protein kinase C, cyclic adenosine monophosphate/protein kinase A, and intracellular pH are known to play important roles in cortical granule exocytosis in eggs and the acrosome reaction in sperm [33,34]. Similar regulatory mechanisms may control the timing of globular body exocytosis and the reversal of undulating membrane propagation in parasperm.

Electron microscopy revealed that globular body contents are released via membrane fusion-mediated exocytosis. Although release is rapid, the contents remain associated with the plasma membrane and do not immediately diffuse. The physiological roles of these materials remain unclear. Proposed functions of parasperm include energy provisioning, modulation of eusperm motility, and inhibition of rival sperm [2,4,5]. The gradual release of globular bodies following copulation suggests that their contents have functional significance during internal fertilization. We observed that eusperm aggregate around residual or released globular body contents, raising the possibility that these materials promote sperm aggregation within inner regions of the female reproductive tract, such as the seminal receptacle (Fig 9B, panel 4). The release of globular body contents may also contribute to sperm competition by interfering with eusperm from rival males. Together with the gradual release of globular bodies, the variation observed in the extent of globular body release and motility suggests that parasperm present in the vas deferens may exist in different physiological states. Differences in the maturation state of parasperm may also represent one aspect of their reproductive strategy. Further in vivo imaging of post-ejaculatory changes in both eusperm and parasperm will be essential for a deeper understanding of parasperm function.

## Conclusion

Eusperm and parasperm are two morphologically distinct types of sperm found in several animal groups. Although parasperm are thought to play important roles in reproductive strategies, their functions and regulatory mechanisms remain poorly understood. In this study, we identified two major changes in the parasperm of the strawberry conch gastropod *Conomurex luhuanus*: the exocytosis of globular bodies and the reversal of the wave propagation direction of the undulating membrane. We demonstrated that globular body exocytosis was suppressed in Ca^2+^ -depleted seawater and promoted either in K^+^ -free seawater or in seawater containing a high concentration of K^+^ . In contrast, reversal of the undulating membrane wave was induced in ASW or by treatment with a Ca^2+^ ionophore, whereas it was suppressed under high extracellular K⁺ conditions, suggesting that these two processes are regulated by distinct mechanisms. These findings provide new insights into the post-ejaculatory behavior of parasperm and their potential functions within the female reproductive tract.

## Supporting information

S1 File Exocytosis of globular body. The image shows a parasperm 30 min after dilution into artificial seawater. An arrow indicates the globular body that undergoes exocytosis. The movie was recorded at 500 fps and played at a speed of 0.06 × . Scale bar, 10 μm.
(MP4)

S2 File Backward swimming of mature parasperm by wave propagation of the undulating membrane. The movie was recorded at 200 fps and played at a speed of 0.15 × . Scale bar, 50 μm.
(MP4)

S1 Table Motility of parasperm under various K^+^ or Ca^2+^ conditions. (XLSX)

S1 Data Underlying data for figures. This excel file contains the minimal data set used to generate Fig. 2B and Figs. 5–8 in the manuscript, with the data for each figure in a separate sheet.
(XLSX)
