# Supplementary material for: Ca2+-dependent exocytosis of parasperm globular bodies in the strawberry conch gastropod Conomurex luhuanus
Source: PLoS One. 2026 Oct 12;21(10):e0360627. doi: 10.1371/journal.pone.0360627 (PMC13657279; doi:10.1371/journal.pone.0360627)
Supplement: Supplementary file 1 [file pone.0360627.s005.docx]

**Reviewer Report – PONE-D-26-23225**

The manuscript “Ca2+-dependent exocytosis of parasperm globular bodies in the gastropod *Conomurex luhuanus*” examines the regulation of globular body exocytosis and motility behavior in parasperm under varying extracellular ionic conditions and pharmacological treatments. The authors aim to clarify the physiological mechanisms underlying parasperm function, with a particular focus on Ca2+ dependence and its relationship to undulating membrane activity.

The study addresses an interesting and still poorly understood aspect of gastropod reproductive biology. The system is potentially valuable, and the manuscript is generally clearly written and easy to follow. The experimental observations are, in principle, relevant and could provide useful insight into post-ejaculatory sperm behavior. However, in its current form, the interpretation of the data often extends beyond what the presented results can robustly support. Several aspects of data structure, statistical treatment, and mechanistic inference require clarification. I therefore consider the study to be potentially suitable for publication after substantial revision and more cautious interpretation of the results.

**Major concerns and recommendations**

**1. Statistical treatment and structure of the data**

The authors base their analyses on 30 individual cells and apply one-way ANOVA followed by Dunnett’s multiple comparison test. While this approach is not inherently inappropriate for small datasets, its validity depends strongly on the underlying distributional assumptions and the independence and representativeness of the sampled observations.

A central issue is that different experimental conditions appear to produce fundamentally different data structures. Under control conditions (e.g., 0 Ca2+ or standard K+), GBFR values are relatively uniform across cells. In contrast, under high Ca2+ conditions, the response appears to split into two distinct populations, with cells showing either minimal exocytosis (GBFR near 100%) or a strong response (GBFR around 25%). This pattern is not compatible with a single homogeneous distribution and suggests condition-dependent heterogeneity in cellular states.

Under these circumstances, the use of mean-based ANOVA comparisons is not sufficient to describe the data. More importantly, summarizing such distributions into a single mean value risks obscuring biologically meaningful subpopulations. The authors should justify the statistical approach in light of these distributional differences and clarify whether assumptions of normality and homoscedasticity were formally tested. Alternative or complementary approaches that reflect the distributional structure should be considered.

**2. Effects of extracellular K+ and interpretation of variability**

The effects of extracellular K+ are described mainly in terms of mean GBFR changes. However, the data indicate that K+ concentration also strongly affects the dispersion of responses.

At low K+ (artificial seawater), GBFR values are widely distributed (approximately 10–100%). With increasing K+ (20–100 mM), there is a progressive narrowing of this distribution, culminating in a highly constrained response range at high K+ (approximately 5–10% at 100 mM). This suggests not only a shift in average exocytotic activity but also a transition toward a more uniform cellular state.

This reduction in variability is not discussed in the manuscript, yet it may be as biologically relevant as the change in mean values. It suggests that extracellular K+ may act as a synchronizing factor for cellular response states rather than simply modulating exocytosis intensity.

In addition, the mechanistic interpretation attributing these effects to membrane potential changes remains speculative. No direct measurements of membrane potential or intracellular K+ levels are provided. This should be clearly acknowledged, and the interpretation should be adjusted accordingly.

**3. Role of Ca2+ and pharmacological evidence**

The conclusion that Ca2+ plays a central role in globular body exocytosis is only partially supported by the presented data. While the absence of extracellular Ca2+ clearly abolishes exocytosis, graded Ca2+ effects are difficult to interpret due to heterogeneous and potentially bimodal responses at higher concentrations. Under these conditions, mean comparisons alone do not capture the structure of the data.

The pharmacological experiments using mibefradil and nifedipine are interpreted as evidence for involvement of T-type Ca2+ channels. However, this interpretation appears premature. Mibefradil is known to lack strict specificity at concentrations commonly used in physiological experiments and may also affect other channels, including Ca2+-activated K+ channels.

Given the strong sensitivity of the system to extracellular K+, an alternative explanation involving indirect modulation of K+-dependent processes cannot be excluded. This possibility is not addressed in the manuscript and should be discussed more explicitly.

Overall, the mechanistic conclusions regarding Ca2+ channel involvement would benefit from a more cautious formulation. Direct measurements of intracellular Ca2+ dynamics and intracellular K+ concentrations would be required to clearly distinguish between direct Ca2+ effects and indirect ionic interactions.

**4. Motility analysis and missing quantitative parameters**

The analysis of parasperm motility is limited to categorical descriptors (forward, backward, immotile). While informative at a basic level, this approach does not capture the full complexity of the observed responses.

Quantitative parameters such as swimming velocity, waveform propagation rate, or beat frequency would substantially strengthen the analysis. This is particularly important given the presence of heterogeneous cellular responses under certain conditions, where distinct functional subpopulations likely exist.

It would also be informative to examine potential correlations between motility patterns, GBFR values, and response heterogeneity. Such an integrated analysis could help determine whether exocytosis state and motility behavior are mechanistically linked or represent independent response pathways.

The current data suggest that high extracellular K+ induces a relatively uniform forward motility state, whereas mibefradil produces a mixed phenotype with immotile and motile subpopulations. Ca2+-free conditions largely abolish motility. These observations raise additional questions regarding the specificity of pharmacological effects and should be discussed more cautiously.

**Revised closing and recommendations (to replace final paragraphs)**

Overall, this manuscript addresses an interesting and potentially important aspect of gastropod reproductive biology. The experimental system is valuable, and the dataset contains observations that could meaningfully advance understanding of parasperm function and ion-dependent regulation of exocytosis and motility.

However, in its current form, the main limitation is not the experimental system itself, but the level of analysis and interpretation. The results indicate clear heterogeneity in cellular responses and strong ion-dependent effects, but these patterns are not sufficiently quantified, integrated, or mechanistically resolved. As a result, several of the central conclusions remain speculative or are not fully supported by the presented data.

For the manuscript to reach the level required for publication, the following issues need to be addressed:

- The statistical analysis must be revised to properly reflect the structure and distribution of the data, including explicit consideration of non-uniform and potentially bimodal response patterns.
- The interpretation of K+-dependent effects requires direct physiological validation. At minimum, intracellular K+ levels or membrane potential changes should be measured under key conditions, including high K+ treatment and mibefradil exposure.
- Ca2+-dependent conclusions should be supported by direct measurements of intracellular Ca2+ dynamics across the relevant experimental conditions, rather than inferred solely from extracellular manipulation.
- Motility analysis should be extended beyond categorical descriptors to include quantitative parameters (e.g., velocity and/or waveform propagation metrics), allowing proper comparison between functional states.
- The apparent presence of distinct cellular subpopulations should be explicitly characterized rather than treated implicitly through mean-based comparisons. Integration of exocytosis and motility data is particularly important to determine whether these represent functionally distinct physiological states.

After these points are addressed and the datasets are integrated in a more quantitative and mechanistically grounded way will it be possible to properly define the biological role of Ca2+ and K+ in regulating parasperm function. With these revisions, the manuscript has strong potential to become a solid and valuable contribution to the field.
