# Supplementary material for: Ca2+-dependent exocytosis of parasperm globular bodies in the strawberry conch gastropod Conomurex luhuanus
Source: PLoS One. 2026 Oct 12;21(10):e0360627. doi: 10.1371/journal.pone.0360627 (PMC13657279; doi:10.1371/journal.pone.0360627)
Supplement: Peer review history

# Peer review: Decision Letter 0

**DOI:** 10.1371/journal.pone.0360627.r001

**Authors:** Sayyed Mohammad Hadi Alavi

21 Jun 2026

Dear Dr. Inaba,

Thank you for submitting your manuscript to PLOS ONE. After careful consideration, we feel that it has merit but does not fully meet PLOS ONE’s publication criteria as it currently stands. Therefore, we invite you to submit a revised version of the manuscript that addresses the points raised during the review process.

Please submit your revised manuscript by Aug 20 2026 11:59PM. If you will need more time than this to complete your revisions, please reply to this message or contact the journal office at plosone@plos.org. When you're ready to submit your revision, log on to https://www.editorialmanager.com/pone/ and select the 'Submissions Needing Revision' folder to locate your manuscript file.

- A letter that responds to each point raised by the academic editor and reviewer(s). You should upload this letter as a separate file labeled 'Response to Reviewers'.
- A marked-up copy of your manuscript that highlights changes made to the original version. You should upload this as a separate file labeled 'Revised Manuscript with Track Changes'.
- An unmarked version of your revised paper without tracked changes. You should upload this as a separate file labeled 'Manuscript'.

If you would like to make changes to your financial disclosure, please include your updated statement in your cover letter. Guidelines for resubmitting your figure files are available below the reviewer comments at the end of this letter.

If applicable, we recommend that you deposit your laboratory protocols in protocols.io to enhance the reproducibility of your results. Protocols.io assigns your protocol its own identifier (DOI) so that it can be cited independently in the future. For instructions see: https://journals.plos.org/plosone/s/submission-guidelines#loc-laboratory-protocols. Additionally, PLOS ONE offers an option for publishing peer-reviewed Lab Protocol articles, which describe protocols hosted on protocols.io. Read more information on sharing protocols at https://plos.org/protocols?utm_medium=editorial-email&utm_source=authorletters&utm_campaign=protocols.

As the corresponding author, your ORCID iD is verified in the submission system and will appear in the published article. PLOS supports the use of ORCID, and we encourage all coauthors to register for an ORCID iD and use it as well. Please encourage your coauthors to verify their ORCID iD within the submission system before final acceptance, as unverified ORCID iDs will not appear in the published article. *Only*  the individual author can complete the verification step; PLOS staff *cannot*  verify ORCID iDs on behalf of authors.

We look forward to receiving your revised manuscript.

Kind regards,

Sayyed Mohammad Hadi Alavi, PhD

Academic Editor

PLOS One

Journal Requirements:

When submitting your revision, we need you to address these additional requirements.

1. Please ensure that your manuscript meets PLOS ONE's style requirements, including those for file naming. The PLOS ONE style templates can be found at

https://journals.plos.org/plosone/s/file?id=wjVg/PLOSOne_formatting_sample_main_body.pdf and https://journals.plos.org/plosone/s/file?id=ba62/PLOSOne_formatting_sample_title_authors_affiliations.pdf

2. In your Methods section, please provide additional information regarding the permits you obtained for the work. Please ensure you have included the full name of the authority that approved the field site access and, if no permits were required, a brief statement explaining why.

3. Please note that PLOS One has specific guidelines on code sharing for submissions in which author-generated code underpins the findings in the manuscript. In these cases, we expect all author-generated code to be made available without restrictions upon publication of the work. Please review our guidelines at https://journals.plos.org/plosone/s/materials-and-software-sharing#loc-sharing-code and ensure that your code is shared in a way that follows best practice and facilitates reproducibility and reuse.

4. Please provide a complete Data Availability Statement in the submission form, ensuring you include all necessary access information or a reason for why you are unable to make your data freely accessible. If your research concerns only data provided within your submission, please write "All data are in the manuscript and/or supporting information files" as your Data Availability Statement.

5. We note that the grant information you provided in the ‘Funding Information’ and ‘Financial Disclosure’ sections do not match.

When you resubmit, please ensure that you provide the correct grant numbers for the awards you received for your study in the ‘Funding Information’ section.

6. Thank you for stating the following financial disclosure:

This work was supported in part by Grants-in-Aid for Scientific Research on Innovative Areas (No. 21112004), Scientific Research on Priority Areas (No. 16083203), Scientific Research B (Nos. 22370023 and 25K02304) and Grant-in-Aid for Transformative Research Areas (No. 21H05304) from the Ministry of Education, Culture, Sports, Science, and Technology, Japan (MEXT) and Japan Society for the Promotion of Science (JSPS). This work was also supported by Sasakawa Scientific Research Grant to RY from the Japan Science Society.

Please state what role the funders took in the study. If the funders had no role, please state: "The funders had no role in study design, data collection and analysis, decision to publish, or preparation of the manuscript."

If this statement is not correct you must amend it as needed.

Please include this amended Role of Funder statement in your cover letter; we will change the online submission form on your behalf.

7. We note that Figure(s) 1, 2, 3, 4, 9, in your submission contain copyrighted images. All PLOS content is published under the Creative Commons Attribution License (CC BY 4.0), which means that the manuscript, images, and Supporting Information files will be freely available online, and any third party is permitted to access, download, copy, distribute, and use these materials in any way, even commercially, with proper attribution. For more information, see our copyright guidelines: http://journals.plos.org/plosone/s/licenses-and-copyright.

We require you to either (1) present written permission from the copyright holder to publish these figures specifically under the CC BY 4.0 license, or (2) remove the figures from your submission:

a. You may seek permission from the original copyright holder of Figure(s) 1, 2, 3, 4, 9, to publish the content specifically under the CC BY 4.0 license.

We recommend that you contact the original copyright holder with the Content Permission Form (http://journals.plos.org/plosone/s/file?id=7c09/content-permission-form.pdf) and the following text:

“I request permission for the open-access journal PLOS ONE to publish XXX under the Creative Commons Attribution License (CCAL) CC BY 4.0 (http://creativecommons.org/licenses/by/4.0/). Please be aware that this license allows unrestricted use and distribution, even commercially, by third parties. Please reply and provide explicit written permission to publish XXX under a CC BY license and complete the attached form.”

Please upload the completed Content Permission Form or other proof of granted permissions as an "Other" file with your submission.

In the figure caption of the copyrighted figure, please include the following text: “Reprinted from [ref] under a CC BY license, with permission from [name of publisher], original copyright [original copyright year].”

b. If you are unable to obtain permission from the original copyright holder to publish these figures under the CC BY 4.0 license or if the copyright holder’s requirements are incompatible with the CC BY 4.0 license, please either i) remove the figure or ii) supply a replacement figure that complies with the CC BY 4.0 license. Please check copyright information on all replacement figures and update the figure caption with source information. If applicable, please specify in the figure caption text when a figure is similar but not identical to the original image and is therefore for illustrative purposes only.

8. If the reviewer comments include a recommendation to cite specific previously published works, please review and evaluate these publications to determine whether they are relevant and should be cited. There is no requirement to cite these works unless the editor has indicated otherwise.

Additional Editor Comments:

Dear Prof. Kazuo Inaba

Thnak you very much for your submission to PLOS ONE. We have completed the peer-review process with the kind helps of three reviewers with expertise in Sperm Biology. They provided very valuable comments that are appended below. I have also found some comments that might be useful to revise the MS.

Thank you for considering PLOS ONE to submit your research.

Very best regards

Hadi Alavi, AE, PLOS ONE

Comments

1) Funding: Please clarify role of funders in the study. If the funders had no role, please state: "The funders had no role in study design, data collection and analysis, decision to publish, or preparation of the manuscript." If this statement is not correct you must amend it as needed.

2) Please add “Authors contribution” and “declaration of competitive interest” at the end of MS before reference.

3- Please let me suggest adding the English name of the organism into the title: in the strawberry conch gastropod (Conomurex luhuanus)

4) Please add a conclusion at the end of the study.

5) Please include number of biological observation and determine the values (mean +/- SD or mean +/- SE) into the legend of Figures 2B, 5, 6 and 7.

6) Please cite the references using numbers.

7) Other comments

L26: the testis, including fertilizing …

L26: This phenomenon termed sperm dimorphism, which is considered to play a role in reproductive strategies such as sperm competition.

L39: These findings indicate that … by different mechanisms. This study provides novel insight into the post-ejaculatory behavior and potential function of parasperm within the female reproductive tract of gastropods.

L52: May be “supportive” as a substitute for “helper”

L59: Please re-write: In the marine sculpin … from rival males (Hayakawa et al., 2002).

L63: It may be useful to add a conclusion from the literature review of parasperm function in fertilization at the end of paragraph. For instance, these studies show biological significance of parasperm in reproduction.

L79: physiological mechanisms … bodies, and found …

L99: It would be useful to indicate the method for identifying gender. It is interesting to know how much sperm does a male produce?

Please provide the methods for “Statistical analysis”

L249: … contents due to the fact that K+ ions is key determinant of sperm membrane potential.

L279: is mainly involved

L330, 337, 359: panels

L369: please expand abbreviation

Figures: Please re-organize the panels in horizontal line for better visualization post type-setting of the article: Panels A, B and C in the first row, and Panels D, E and F in the second row.

[Note: HTML markup is below. Please do not edit.]

Reviewers' comments:

Reviewer's Responses to Questions

**Comments to the Author**

1. Is the manuscript technically sound, and do the data support the conclusions?

Reviewer #1: Yes

Reviewer #2: Partly

Reviewer #3: Partly

**********

2. Has the statistical analysis been performed appropriately and rigorously?

Reviewer #1: Yes

Reviewer #2: Yes

Reviewer #3: No

**********

3. Have the authors made all data underlying the findings in their manuscript fully available?

The PLOS Data policy

Reviewer #1: Yes

Reviewer #2: Yes

Reviewer #3: Yes

**********

4. Is the manuscript presented in an intelligible fashion and written in standard English?

Reviewer #1: Yes

Reviewer #2: Yes

Reviewer #3: Yes

**********

Reviewer #1: The manuscript describes Ca2+-dependent exocytosis of globular bodies in parasperm of Conomurex luhuanus and compares this process with regulation of undulating membrane motility. The observations are interesting and the study provides useful descriptive and mechanistic information on an understudied reproductive cell type. Overall, the manuscript appears suitable for publication after revision, but several interpretations should be toned down or better supported.

Major comments

1.The claim that Ca2+ influx “triggers” globular body exocytosis should be moderated.

Ca2+-free ASW suppresses exocytosis, and mibefradil inhibits the process, supporting a role for Ca2+. However, high extracellular Ca2+ and ionomycin do not further enhance exocytosis. Therefore, the data support the conclusion that Ca2+ is required or involved, but not necessarily that Ca2+ influx alone triggers exocytosis. The authors should revise the wording accordingly.

2.The interpretation of T-type Ca2+ channel involvement relies heavily on pharmacology.

Mibefradil strongly suppresses globular body exocytosis and motility, but mibefradil can have off-target effects, especially at higher concentrations. The authors should acknowledge this limitation and avoid presenting the result as definitive proof of T-type Ca2+ channel function.

Minor comments

1.Abstract: “was not even enhanced by very high Ca2+ conditions” should be revised for clarity, e.g. “was not further enhanced by high extracellular Ca2+ or ionomycin treatment.”

2.Introduction: lack of introduction of ionic effect on sperm motility.

3.Statistical analysis: please state how the assumptions of ANOVA were assessed, including normality of residuals and homogeneity of variance. If these assumptions were not met, data transformation or appropriate non-parametric/robust alternatives should be considered.

4.“Remain poorly understood at the molecular mechanism” should be changed to “remain poorly understood at the mechanistic level.”

5.Correct typographical errors such as “ware observed” and “ware induced” to “were observed” and “were induced.”

6.“Per a cell” should be changed to “per cell.”

7.“Two lateral undulating membrane” should be “two lateral undulating membranes.”

8.“K+-dependent membrane potential play an important role” should be “plays an important role.”

9.“Alternations in membrane potential” should be “alterations in membrane potential.”

10.The Fig. 1 legend appears to refer to panels F and G, although only panels A-E are described. This should be checked.

11.The term “globular body filling rate” should be more clearly defined when first introduced.

12.The authors should clarify whether Ca2+-free ASW contained a Ca²⁺ chelator such as EGTA, or whether CaCl₂ was simply omitted.

13.Exact p values would be preferable, or at least the statistical reporting should be made more complete.

14.The number of cells, individuals, and sections examined in TEM should be stated.

15.Formatting of Ca²+, K+, CaCl₂, KCl, and units should be standardized throughout the manuscript.

Reviewer #2: This interesting study shows that the globular body observed in parasperm is released via exocytosis, verified through TEM and live observation. The phenomenon itself is convincing. On the other hand, many of these key conclusions are derived from only limited pharmacological experiments, and it appears that speculation currently predominates regarding the molecular nature of the Ca²⁺ channels, the K⁺-dependent regulatory mechanisms, and their physiological roles within the reproductive tract. In particular, the model presented in the Discussion section and Figure 9 does not appear to be fully supported by the current data.

In conclusion, while the data presented in this manuscript are of high value and show potential, it is necessary to more clearly distinguish between what can be definitively stated based on these findings and the speculative elements, and to reorganize the manuscript before acceptance.

Specific points

1) One of the key questions in this study is why the parasperm releases globular bodies. However, current understanding of their function remains insufficient. More in-depth analysis is needed regarding the behavior of the released contents and their relationship to the eusperm.

Is it possible to discuss, in particular, whether the released material might be involved in the dissociation or subsequent reaggregation of eusperm bundles?

2) Since exocytosis is inhibited in the absence of Ca²⁺, the conclusion that Ca²⁺ is necessary for the exocytosis of globular bodies is valid. However, I find it necessary to examine more carefully whether Ca²⁺ actually acts as a trigger for the exocytosis.

As shown in Figure 6, exocytosis occurs under normal seawater conditions, whereas additional enhancement is observed only under elevated Ca²⁺ concentrations (20 mM or higher). First, high Ca²⁺ conditions of 40–100 mM are extremely high compared to typical seawater environments and could hardly be considered physiological concentrations. Even at 20 mM, this is double the concentration of seawater. Considering that Ca²⁺ concentrations in seawater are generally already quite high compared to intracellular levels, it is questionable whether environments with Ca²⁺ concentrations higher than those in seawater actually exist. Figure 9 presents a model based on the assumption that a high Ca²⁺ environment exists within the female reproductive tract; however, if the authors intend to maintain this model, additional evidence—such as actual measurements of Ca²⁺ concentrations in reproductive tract fluid—is required. Otherwise, it should be presented as a more hypothetical model.

Furthermore, ionomycin does not induce exocytosis, suggesting that Ca²⁺ alone is not sufficient and that additional signaling pathways may be involved in triggering exocytosis. This point should be more thoroughly discussed in the Discussion section.

3) It is interesting that this exocytosis is sensitive to mibefradil but insensitive to nifedipine. However, the current data only support the existence of a “mibefradil-sensitive Ca²⁺ influx pathway”; there is insufficient evidence to conclude that its molecular identity is a T-type Ca²⁺ channel. In particular, CatSper, a sperm-specific Ca²⁺ channel well known in mammals, is known to be sensitive to mibefradil. Although CatSper itself is not believed to exist in protostomes, the possibility of an unknown mibefradil-sensitive Ca²⁺ influx pathway should also be considered. Given the possibility that Ca²⁺ alone does not induce exocytosis, it is conceivable that other signaling pathways, including intracellular pH, are involved. While I do not require additional experiments, I believe it would be worthwhile to mention this possibility in the Discussion section.

Furthermore, it is not self-evident that invertebrates share the same pharmacological characteristics as vertebrates; therefore, careful examination and discussion are necessary, taking into account the action characteristics of the agents and amino acid sequence homology.

4) Regarding K⁺-dependent regulation, it appears to be the most problematic aspect of this study. Since exocytosis was promoted under both K⁺-deficient and high-K⁺ conditions, the current data alone do not support determining whether depolarization or hyperpolarization is the key factor, and thus the conclusion that “exocytosis is regulated by changes in membrane potential” is not sufficiently supported by the current data.

I recommend that the authors conduct additional experiments, such as measuring membrane potential changes using fluorescent probes, to verify these findings. Nonetheless, at this stage, these results do not clarify the underlying mechanism, and the tone of the Discussion section should be significantly toned down.

5) Figure 9 presents an attractive model. As described above, however, several key assumptions, including the existence of high-Ca²⁺ and high-K⁺ environments in the female reproductive tract, remain untested. These assumptions should either be experimentally supported or more clearly presented as hypothetical elements of the model.

6) The authors should clarify the number of biological replicates used in each experiment. Because multiple cells appear to have been analyzed from a limited number of individuals, it would be helpful to explain how pseudoreplication was avoided in the statistical analyses.

Reviewer #3: Reviewer Report – PONE-D-26-23225

The manuscript “Ca2+-dependent exocytosis of parasperm globular bodies in the gastropod Conomurex luhuanus” examines the regulation of globular body exocytosis and motility behavior in parasperm under varying extracellular ionic conditions and pharmacological treatments. The authors aim to clarify the physiological mechanisms underlying parasperm function, with a particular focus on Ca2+ dependence and its relationship to undulating membrane activity.

The study addresses an interesting and still poorly understood aspect of gastropod reproductive biology. The system is potentially valuable, and the manuscript is generally clearly written and easy to follow. The experimental observations are, in principle, relevant and could provide useful insight into post-ejaculatory sperm behavior. However, in its current form, the interpretation of the data often extends beyond what the presented results can robustly support. Several aspects of data structure, statistical treatment, and mechanistic inference require clarification. I therefore consider the study to be potentially suitable for publication after substantial revision and more cautious interpretation of the results.

Major concerns and recommendations

1. Statistical treatment and structure of the data

The authors base their analyses on 30 individual cells and apply one-way ANOVA followed by Dunnett’s multiple comparison test. While this approach is not inherently inappropriate for small datasets, its validity depends strongly on the underlying distributional assumptions and the independence and representativeness of the sampled observations.

A central issue is that different experimental conditions appear to produce fundamentally different data structures. Under control conditions (e.g., 0 Ca2+ or standard K+), GBFR values are relatively uniform across cells. In contrast, under high Ca2+ conditions, the response appears to split into two distinct populations, with cells showing either minimal exocytosis (GBFR near 100%) or a strong response (GBFR around 25%). This pattern is not compatible with a single homogeneous distribution and suggests condition-dependent heterogeneity in cellular states.

Under these circumstances, the use of mean-based ANOVA comparisons is not sufficient to describe the data. More importantly, summarizing such distributions into a single mean value risks obscuring biologically meaningful subpopulations. The authors should justify the statistical approach in light of these distributional differences and clarify whether assumptions of normality and homoscedasticity were formally tested. Alternative or complementary approaches that reflect the distributional structure should be considered.

2. Effects of extracellular K+ and interpretation of variability

The effects of extracellular K+ are described mainly in terms of mean GBFR changes. However, the data indicate that K+ concentration also strongly affects the dispersion of responses.

At low K+ (artificial seawater), GBFR values are widely distributed (approximately 10–100%). With increasing K+ (20–100 mM), there is a progressive narrowing of this distribution, culminating in a highly constrained response range at high K+ (approximately 5–10% at 100 mM). This suggests not only a shift in average exocytotic activity but also a transition toward a more uniform cellular state.

This reduction in variability is not discussed in the manuscript, yet it may be as biologically relevant as the change in mean values. It suggests that extracellular K+ may act as a synchronizing factor for cellular response states rather than simply modulating exocytosis intensity.

In addition, the mechanistic interpretation attributing these effects to membrane potential changes remains speculative. No direct measurements of membrane potential or intracellular K+ levels are provided. This should be clearly acknowledged, and the interpretation should be adjusted accordingly.

3. Role of Ca2+ and pharmacological evidence

The conclusion that Ca2+ plays a central role in globular body exocytosis is only partially supported by the presented data. While the absence of extracellular Ca2+ clearly abolishes exocytosis, graded Ca2+ effects are difficult to interpret due to heterogeneous and potentially bimodal responses at higher concentrations. Under these conditions, mean comparisons alone do not capture the structure of the data.

The pharmacological experiments using mibefradil and nifedipine are interpreted as evidence for involvement of T-type Ca2+ channels. However, this interpretation appears premature. Mibefradil is known to lack strict specificity at concentrations commonly used in physiological experiments and may also affect other channels, including Ca2+-activated K+ channels.

Given the strong sensitivity of the system to extracellular K+, an alternative explanation involving indirect modulation of K+-dependent processes cannot be excluded. This possibility is not addressed in the manuscript and should be discussed more explicitly.

Overall, the mechanistic conclusions regarding Ca2+ channel involvement would benefit from a more cautious formulation. Direct measurements of intracellular Ca2+ dynamics and intracellular K+ concentrations would be required to clearly distinguish between direct Ca2+ effects and indirect ionic interactions.

4. Motility analysis and missing quantitative parameters

The analysis of parasperm motility is limited to categorical descriptors (forward, backward, immotile). While informative at a basic level, this approach does not capture the full complexity of the observed responses.

Quantitative parameters such as swimming velocity, waveform propagation rate, or beat frequency would substantially strengthen the analysis. This is particularly important given the presence of heterogeneous cellular responses under certain conditions, where distinct functional subpopulations likely exist.

It would also be informative to examine potential correlations between motility patterns, GBFR values, and response heterogeneity. Such an integrated analysis could help determine whether exocytosis state and motility behavior are mechanistically linked or represent independent response pathways.

The current data suggest that high extracellular K+ induces a relatively uniform forward motility state, whereas mibefradil produces a mixed phenotype with immotile and motile subpopulations. Ca2+-free conditions largely abolish motility. These observations raise additional questions regarding the specificity of pharmacological effects and should be discussed more cautiously.

Revised closing and recommendations (to replace final paragraphs)

Overall, this manuscript addresses an interesting and potentially important aspect of gastropod reproductive biology. The experimental system is valuable, and the dataset contains observations that could meaningfully advance understanding of parasperm function and ion-dependent regulation of exocytosis and motility.

However, in its current form, the main limitation is not the experimental system itself, but the level of analysis and interpretation. The results indicate clear heterogeneity in cellular responses and strong ion-dependent effects, but these patterns are not sufficiently quantified, integrated, or mechanistically resolved. As a result, several of the central conclusions remain speculative or are not fully supported by the presented data.

For the manuscript to reach the level required for publication, the following issues need to be addressed:

• The statistical analysis must be revised to properly reflect the structure and distribution of the data, including explicit consideration of non-uniform and potentially bimodal response patterns.

• The interpretation of K+-dependent effects requires direct physiological validation. At minimum, intracellular K+ levels or membrane potential changes should be measured under key conditions, including high K+ treatment and mibefradil exposure.

• Ca2+-dependent conclusions should be supported by direct measurements of intracellular Ca2+ dynamics across the relevant experimental conditions, rather than inferred solely from extracellular manipulation.

• Motility analysis should be extended beyond categorical descriptors to include quantitative parameters (e.g., velocity and/or waveform propagation metrics), allowing proper comparison between functional states.

• The apparent presence of distinct cellular subpopulations should be explicitly characterized rather than treated implicitly through mean-based comparisons. Integration of exocytosis and motility data is particularly important to determine whether these represent functionally distinct physiological states.

After these points are addressed and the datasets are integrated in a more quantitative and mechanistically grounded way will it be possible to properly define the biological role of Ca2+ and K+ in regulating parasperm function. With these revisions, the manuscript has strong potential to become a solid and valuable contribution to the field.

**********

what does this mean?). If published, this will include your full peer review and any attached files.

If you choose “no”, your identity will remain anonymous but your review may still be made public.

**Do you want your identity to be public for this peer review?**  For information about this choice, including consent withdrawal, please see our Privacy Policy

Reviewer #1: No

Reviewer #2: No

Reviewer #3: No

**********

[NOTE: If reviewer comments were submitted as an attachment file, they will be attached to this email and accessible via the submission site. Please log into your account, locate the manuscript record, and check for the action link "View Attachments". If this link does not appear, there are no attachment files.]

To ensure your figures meet our technical requirements, please review our figure guidelines: https://journals.plos.org/plosone/s/figures

You may also use PLOS’s free figure tool, NAAS, to help you prepare publication quality figures: https://journals.plos.org/plosone/s/figures#loc-tools-for-figure-preparation.

NAAS will assess whether your figures meet our technical requirements by comparing each figure against our figure specifications.

Attachment Submitted filename: PONE.docx

# Author comment: Author response to Decision Letter 1

**DOI:** 10.1371/journal.pone.0360627.r002

10 Sep 2026

Response to Reviewers:

For clarity, the response is indicated in blue here and in the revised manuscript

Comments by editor

1) Funding: Please clarify role of funders in the study. If the funders had no role, please state: "The funders had no role in study design, data collection and analysis, decision to publish, or preparation of the manuscript." If this statement is not correct you must amend it as needed.

(response)

We have modified the description for Funding in the MS submission system as following:

This work was supported in part by Grants-in-Aid for Scientific Research on Innovative Areas (No. 21112004), Scientific Research on Priority Areas (No. 16083203), Scientific Research B (Nos. 22370023 and 25K02304) and Grant-in-Aid for Transformative Research Areas (No. 21H05304) from the Ministry of Education, Culture, Sports, Science, and Technology, Japan (MEXT) and Japan Society for the Promotion of Science (JSPS). This work was also supported by Sasakawa Scientific Research Grant to RY from the Japan Science Society. These grants played no role in the study design, data collection, analysis, decision to publish, or preparation of the manuscript.

2) Please add “Authors contribution” and “declaration of competitive interest” at the end of MS before reference.

(response)

We have added “Author contribution” and “declaration of competitive interest” at the end of our MS. (Line 477-489)

Author Contributions

Conceptualization: Kazuo Inaba.

Sample collection: Ryo Yokoya, Masaya Morita.

Data curation: Ryo Yokoya, Kogiku Shiba, Masaya Morita.

Formal analysis: Ryo Yokoya.

Investigation: Ryo Yokoya.

Methodology: Ryo Yokoya, Kogiku Shiba, Masaya Morita, Kazuo Inaba.

Project administration: Kazuo Inaba.

Supervision: Kazuo Inaba.

Validation: Ryo Yokoya, Kogiku Shiba, Masaya Morita, Kazuo Inaba.

Visualization: Ryo Yokoya, Kogiku Shiba.

Writing – original draft: Ryo Yokoya, Kazuo Inaba.

Writing – review & editing: Ryo Yokoya, Kogiku Shiba, Masaya Morita, Kazuo Inaba.

Declaration of competitive interest:

The authors have declared that no competing interests exist. (Line 491-492)

3) Please let me suggest adding the English name of the organism into the title: in the strawberry conch gastropod (Conomurex luhuanus)

(response)

According to the editor’s suggestion, the title has been changed as follows:

Ca2+-dependent exocytosis of parasperm globular bodies in the strawberry conch gastropod Conomurex luhuanus (Title page)

4) Please add a conclusion at the end of the study.

(response)

We have now added a conclusion at the end of manuscript. (Line 459-471)

5) Please include number of biological observation and determine the values (mean ± SD or mean ± SE) into the legend of Figures 2B, 5, 6 and 7.

(response)

In the revised manuscript, we performed GLMM analysis. As suggested in the comment, we have added the number of samples and observation and indicated overall means and 95% confidence intervals in each figure legend. According to the comment by reviewer 3, we have revised Fig. 8 and thereby also shown mean ± SD values for Fig. 8 separately in Table 1. (Line 221-228; 290-298; 312-321; 338-348)

6) Please cite the references using numbers.

(response)

We have changed the citation format in the text to numbered references.

7) Other comments

L26: the testis, including fertilizing …

(response)

Corrected as suggested.

L26: This phenomenon termed sperm dimorphism, which is considered to play a role in reproductive strategies such as sperm competition.

(response)

Corrected as suggested.

L39: These findings indicate that … by different mechanisms. This study provides novel insight into the post-ejaculatory behavior and potential function of parasperm within the female reproductive tract of gastropods.

(response)

Corrected as suggested.

L52: May be “supportive” as a substitute for “helper”

(response)

Corrected as suggested.

L59: Please re-write: In the marine sculpin … from rival males (Hayakawa et al., 2002).

(response)

We regret that the sentence was not clear enough. We have changed it as follows:

In the marine sculpin Hemilepidotus gilberti, parasperm released into seawater form cellular aggregates on the surface of egg masses that capture eusperm from rival males and inhibit their motility. (Line 57-59)

L63: It may be useful to add a conclusion from the literature review of parasperm function in fertilization at the end of paragraph. For instance, these studies show biological significance of parasperm in reproduction.

(response)

We have added a sentence as follows:

Collectively, these studies demonstrate the biological significance of parasperm in reproduction. (Line 61-62)

L79: physiological mechanisms … bodies, and found …

(response)

Corrected as suggested.

L99: It would be useful to indicate the method for identifying gender. It is interesting to know how much sperm does a male produce?

Please provide the methods for “Statistical analysis”

(response)

We have added the following sentences:

Sex was determined based on the presence or absence of a protruding penis on the right side of the head-foot region. During the peak of the reproductive season, up to 20 μL of semen could be collected from each male. (Line 107-110)

Methods for Statistical analysis have been added at the end of “Materials and methods” section.

L249: … contents due to the fact that K+ ions is key determinant of sperm membrane potential.

(response)

Corrected as suggested.

L279: is mainly involved

(response)

Corrected as suggested.

L330, 337, 359: panels

(response)

Corrected as suggested.

L369: please expand abbreviation

(response)

Corrected as suggested.

Figures: Please re-organize the panels in horizontal line for better visualization post type-setting of the article: Panels A, B and C in the first row, and Panels D, E and F in the second row.

(response)

As suggested, we revised the layouts of Figures 1, 2, and 4. For Figure 3, arranging the panels side by side results in an unbalanced overall appearance, so we would like to retain the original layout.

To Editor:

In response to the request from the Editorial Manager, we have added information regarding the copyright of the images in the manuscript submission system. We have also added S1 Data as Supporting Information and provided descriptions of this dataset in the Materials and Methods (Statistical analysis) and in the figure legends for Fig. 2B and Figs. 5–8.

Reviewers' comments:

Reviewer #1:

The manuscript describes Ca2+-dependent exocytosis of globular bodies in parasperm of Conomurex luhuanus and compares this process with regulation of undulating membrane motility. The observations are interesting and the study provides useful descriptive and mechanistic information on an understudied reproductive cell type. Overall, the manuscript appears suitable for publication after revision, but several interpretations should be toned down or better supported.

Major comments

1.The claim that Ca2+ influx “triggers” globular body exocytosis should be moderated.

Ca2+-free ASW suppresses exocytosis, and mibefradil inhibits the process, supporting a role for Ca2+. However, high extracellular Ca2+ and ionomycin do not further enhance exocytosis. Therefore, the data support the conclusion that Ca2+ is required or involved, but not necessarily that Ca2+ influx alone triggers exocytosis. The authors should revise the wording accordingly.

(response)

We agree that, based on several of the data, it is not appropriate to state definitively that Ca2+ acts as the trigger for globular body exocytosis, as the reviewer points out. We have therefore removed the term “trigger” and softened the wording accordingly.

On the other hand, as also reflected in our responses to the other comments, based on the results obtained with ionomycin, we performed additional experiments using another Ca2+ ionophore, A23187. These experiments provided stronger support for a requirement for Ca2+. The results also suggested that ionomycin may not have been acting effectively under our experimental conditions, while higher concentrations caused substantial toxicity. We therefore replaced the ionomycin results with the results obtained using A23187 and revised the corresponding descriptions accordingly. (Line 304; 307-310; 316-325; 370-371; 388-391)

2.The interpretation of T-type Ca2+ channel involvement relies heavily on pharmacology.

Mibefradil strongly suppresses globular body exocytosis and motility, but mibefradil can have off-target effects, especially at higher concentrations. The authors should acknowledge this limitation and avoid presenting the result as definitive proof of T-type Ca2+ channel function.

(response)

At present, we have no alternative but to rely on the data obtained from pharmacological experiments. In response to the reviewer’s comment, we have added data obtained using another T-type channel inhibitor, NNC55-0396. Treatment with this inhibitor also suppressed globular body exocytosis. Accordingly, we have softened the wording regarding the involvement of T-type channels. (Line 331; 336-338)

Minor comments

1.Abstract: “was not even enhanced by very high Ca2+ conditions” should be revised for clarity, e.g. “was not further enhanced by high extracellular Ca2+ or ionomycin treatment.”

(response)

We thank the reviewer for pointing this out. Because we had concerns about the results obtained with ionomycin, we performed additional experiments using A23187 during the revision process. Based on these new results, we have revised the corresponding text accordingly. (Line 304; 307-310; 316-325; 370-371; 388-391)

2.Introduction: lack of introduction of ionic effect on sperm motility.

(response)

We thank the reviewer for this comment. We have added a description of ion regulation in eusperm to the final paragraph of the Introduction. (Line 75-81)

3.Statistical analysis: please state how the assumptions of ANOVA were assessed, including normality of residuals and homogeneity of variance. If these assumptions were not met, data transformation or appropriate non-parametric/robust alternatives should be considered.

(Response)

We have substantially revised the statistical analyses. Rather than using ANOVA, the proportion data were analyzed using beta-binomial generalized linear mixed models (GLMMs), because overdispersion was detected in the binomial models. Individual identity was included as a random effect to account for the non-independence of repeated observations obtained from the same individual, thereby avoiding pseudoreplication. Model adequacy and residual dispersion were evaluated using simulation-based residual diagnostics (DHARMa). Where residual dispersion differed among treatments, treatment-specific dispersion was incorporated into the beta-binomial model.

Model-estimated proportions and 95% confidence intervals (CIs) were calculated, and comparisons with the control were performed using Dunnett-adjusted multiple comparisons. (Line 155-175)

As a result of this substantial revision of the statistical analysis, we have generated new figures for Fig. 2B, Fig. 5, Fig. 6, and Fig. 7. (Line 223-230; 293-301; 316-325; 343-353)

4.“Remain poorly understood at the molecular mechanism” should be changed to “remain poorly understood at the mechanistic level.”

(response)

We thank the reviewer for this valuable comment. We felt that the term “mechanistic” did not accurately convey the point we intended to make. We have therefore revised the wording to “their behavior and function remain poorly understood at the molecular level.” (Line 49-50; 468-469)

5.Correct typographical errors such as “ware observed” and “ware induced” to “were observed” and “were induced.”

(response)

Corrected as suggested.

6.“Per a cell” should be changed to “per cell.”

(response)

Corrected as suggested.

7.“Two lateral undulating membrane” should be “two lateral undulating membranes.”

(response)

Corrected as suggested.

8.“K+-dependent membrane potential play an important role” should be “plays an important role.”

(response)

Corrected as suggested.

9.“Alternations in membrane potential” should be “alterations in membrane potential.”

(response)

Corrected as suggested.

10.The Fig. 1 legend appears to refer to panels F and G, although only panels A-E are described. This should be checked.

(response)

This was an oversight on our part. We have corrected it accordingly.

11.The term “globular body filling rate” should be more clearly defined when first introduced.

(response)

We have added a detailed description of how the appearance of globular bodies and the globular body filling rate were determined. (Line 204-211)

12.The authors should clarify whether Ca2+-free ASW contained a Ca²⁺ chelator such as EGTA, or whether CaCl₂ was simply omitted.

(response)

Ca-free seawater refers to calcium-free seawater supplemented with EGTA. We have added this information to the Materials and Methods section. (Line 93-95)

13.Exact p values would be preferable, or at least the statistical reporting should be made more complete.

(Response)

According to the comment, we have now indicated exact p values in Figs. 5, 6 and 7.

14.The number of cells, individuals, and sections examined in TEM should be stated.

(response)

The samples used for TEM analysis were obtained from multiple preparations, each of which contained a large number of parasperm. We examined up to 43 cells in total. Of these, 23 cells were examined and documented in detail, and representative cells from high-quality samples with typical morphology are presented in the figures. (Line 275-277)

15.Formatting of Ca²+, K+, CaCl₂, KCl, and units should be standardized throughout the manuscript.

(response)

We have revised the text to consistently use “CaCl₂” and “KCl” when referring to the composition of solutions used in the experiments, and “Ca²⁺” and “K⁺” when describing the general actions of these ions.

Reviewer #2:

This interesting study shows that the globular body observed in parasperm is released via exocytosis, verified through TEM and live observation. The phenomenon itself is convincing. On the other hand, many of these key conclusions are derived from only limited pharmacological experiments, and it appears that speculation currently predominates regarding the molecular nature of the Ca²⁺ channels, the K⁺-dependent regulatory mechanisms, and their physiological roles within the reproductive tract. In particular, the model presented in the Discussion section and Figure 9 does not appear to be fully supported by the current data.

In conclusion, while the data presented in this manuscript are of high value and show potential, it is necessary to more clearly distinguish between what can be definitively stated based on these findings and the speculative elements, and to reorganize the manuscript before acceptance.

Specific points

1) One of the key questions in this study is why the parasperm releases globular bodies. However, current understanding of their function remains insufficient. More in-depth analysis is needed regarding the behavior of the released contents and their relationship to the eusperm.

Is it possible to discuss, in particular, whether the released material might be involved in the dissociation or subsequent reaggregation of eusperm bundles?

(Response)

The point raised by the reviewer is indeed one of the important questions that needs to be addressed to better understand the biology of parasperm. Because we observed an aggregation phenomenon, we have described this observation in the present manuscript and have incorporated a brief discussion of its possible implications. (Line 455-464)

The biochemical characterization of the globular bodies is currently underway, and we hope to report these findings in a separate study in the future.

2) Since exocytosis is inhibited in the absence of Ca²⁺, the conclusion that Ca²⁺ is necessary for the exocytosis of globular bodies is valid. However, I find it necessary to examine more carefully whether Ca²⁺ actually acts as a trigger for the exocytosis.

As shown in Figure 6, exocytosis occurs under normal seawater conditions, whereas additional enhancement is observed only under elevated Ca²⁺ concentrations (20 mM or higher). First, high Ca²⁺ conditions of 40–100 mM are extremely high compared to typical seawater environments and could hardly be considered physiological concentrations. Even at 20 mM, this is double the concentration of seawater. Considering that Ca²⁺ concentrations in seawater are generally already quite high compared to intracellular levels, it is questionable whether environments with Ca²⁺ concentrations higher than those in seawater actually exist. Figure 9 presents a model based on the assumption that a high Ca²⁺ environment exists within the female reproductive tract; however, if the authors intend to maintain this model, additional evidence—such as actual measurements of Ca²⁺ concentrations in reproductive tract fluid—is required. Otherwise, it should be presented as a more hypothetical model.

Furthermore, ionomycin does not induce exocytosis, suggesting that Ca²⁺ alone is not sufficient and that additional signaling pathways may be involved in triggering exocytosis. This point should be more thoroughly discussed in the Discussion section.

(Response)

We have also considered imaging of intracellular Ca2+ and membrane potential as approaches to more directly investigate the dynamics of intracellular ions. However, particularly for in vivo imaging, there are many technical challenges that remain to be overcome, and we have not yet been able to implement these approaches.

Regarding Ca2+, in this revision we have added new results obtained using A23187, as the issues observed with ionomycin may have been related to its toxicity. In addition, we used another T-type channel inhibitor, NNC55-0396, and obtained results that further support the involvement of T-type channels. (Line 304; 307-310; 316-325; 370-371; 388-391)

In the present manuscript, we have included Fig. 9A and B as a hypothetical model. We have slightly softened the wording to avoid overstatement and have added several descriptions to further explain and support the proposed model.

3) It is interesting that this exocytosis is sensitive to mibefradil but insensitive to nifedipine. However, the current data only support the existence of a “mibefradil-sensitive Ca²⁺ influx pathway”; there is insufficient evidence to conclude that its molecular identity is a T-type Ca²⁺ channel. In particular, CatSper, a sperm-specific Ca²⁺ channel well known in mammals, is known to be sensitive to mibefradil. Although CatSper itself is not believed to exist in protostomes, the possibility of an unknown mibefradil-sensitive Ca²⁺ influx pathway should also be considered. Given the possibility that Ca²⁺ alone does not induce exocytosis, it is conceivable that other signaling pathways, including intracellular pH, are involved. While I do not require additional experiments, I believe it would be worthwhile to mention this possibility in the Discussion section.

Furthermore, it is not self-evident that invertebrates share the same pharmacological characteristics as vertebrates; therefore, careful examination and discussion are necessary, taking into account the action characteristics of the agents and amino acid sequence homology.

(Response)

We agree with the reviewer that the mechanisms involved in parasperm may not necessarily be the same as those in vertebrates. We also acknowledge that other signaling pathways may be involved and have added a discussion of their possible involvement in the revised manuscript based on the available literature. (Line 439-440)

Unfortunately, no genomic information is currently available for this species that would allow us to perform a molecular comparison with vertebrates. Therefore, it is difficult to fully address this question at the molecular level. Nevertheless, in this revision, we have added new results obtained using another T-type channel inhibitor, NNC55-0396. The inhibitory effect of NNC55-0396 on globular body exocytosis provides additional support for the involvement of T-type Ca2+ channels. (Line 331; 336-338)

4) Regarding K⁺-dependent regulation, it appears to be the most problematic aspect of this study. Since exocytosis was promoted under both K⁺-deficient and high-K⁺ conditions, the current data alone do not support determining whether depolarization or hyperpolarization is the key factor, and thus the conclusion that “exocytosis is regulated by changes in membrane potential” is not sufficiently supported by the current data.

I recommend that the authors conduct additional experiments, such as measuring membrane potential changes using fluorescent probes, to verify these findings. Nonetheless, at this stage, these results do not clarify the underlying mechanism, and the tone of the Discussion section should be significantly toned down.

(Response)

As the reviewer points out, this is one of the most puzzling aspects of our results, and we also find it difficult to interpret. At higher K+ concentrations, depolarization occurs, and we speculate that voltage-dependent Ca2+ channels, such as those found in neurons and other excitable cells, may be involved. This possibility is also related to our discussion that K+ concentrations may increase within the female reproductive tract. (Line 439-440)

A remaining question, however, is why globular body exocytosis is also activated at low K+ concentrations, which would be expected to cause depolarization under our experimental conditions. Similar responses to low K+ conditions are known as part of the activation mechanisms of many types of sperm. Nevertheless, as the reviewer points out, experiments using fluorescent probes could provide important insights into the underlying ionic dynamics. Because this is a cell type and organism for which there is currently little experimental background for such approaches, however, establishing these methods will require considerable time. We therefore consider this an important topic for future investigation.

Regarding the description in the Discussion, we have slightly toned down our statements to avoid overstating the interpretation.

5) Figure 9 presents an attractive model. As described above, however, several key assumptions, including the existence of high-Ca²⁺ and high-K⁺ environments in the female reproductive tract, remain untested. These assumptions should either be experimentally supported or more clearly presented as hypothetical elements of the model.

(Response)

As the reviewer points out, we agree that measurements of the detailed ionic environment within the female reproductive tract would be important for understanding the physiological relevance of our findings. However, as described in our response to Comment 2, in vivo imaging remains technically challenging at present, and we consider this an important subject for future investigation.

For the present study, we would therefore like to limit our conclusions to the results obtained using A23187 and NNC55-0396 and their support for our proposed hypothesis. (Line 304; 307-310; 316-325; 370-371; 388-391)

6) The authors should clarify the number of biological replicates used in each experiment. Because multiple cells appear to have been analyzed from a limited number of individuals, it would be helpful to explain how pseudoreplication was avoided in the statistical analyses.

(Response)

Thank you for pointing this out. We have now clarified the number of biological replicates used in each experiment in the revised manuscript. Although we analyzed multiple cells from each individual, we did not treat these observations as independent biological replicates. Instead, we included individual identity as a random effect in the generalized linear mixed models (GLMMs), accounting for the non-independence of repeated observations from the same individual and avoiding pseudoreplication.

As also noted in our responses to the other statistical comments, atypical sperm showed substantial variability among individuals and experimental conditions. We therefore substantially revised the statistical analyses. For the Ca2+ experiments, we used beta-binomial GLMMs and incorporated treatment-specific dispersion where residual variability differed among Ca2+ concentrations. For the inhibitor experiments, we also used beta-binomial GLMMs and incorporated additional data not included in the original manuscript into the revised analyses. We compared results with the ASW control using Dunnett-adjusted multiple comparisons.

The revised manuscript and figure legends now explicitly report the number of biological replicates and analyzed observations for each experiment. (Line 155-175; 224-231; 293-301; 316-325; 343-353)

Reviewer #3:

Reviewer Report – PONE-D-26-23225

The manuscript “Ca2+-dependent exocytosis of parasperm globular bodies in the gastropod Conomurex luhuanus” examines the regulation of globular body exocytosis and motility behavior in parasperm under varying extracellular ionic conditions and pharmacological treatments. The authors aim to clarify the physiological mechanisms underlying parasperm function, with a particular focus on Ca2+ dependence and its relationship to undulating membrane activity.

The study addresses an interesting and still poorly understood aspect of gastropod reproductive biology. The system is potentially valuable, and the manuscript is generally clearly written and easy to follow. The experimental observations are, in principle, relevant and could provide useful insight into post-ejaculatory sperm behavior. However, in its current form, the interpretation of the data often extends beyond what the presented results can robustly support. Several aspects of data structure, statistical treatment, and mechanistic inference require clarification. I therefore consider the study to be potentially suitable for publication after substantial revision and more cautious interpretation of the results.

Major concerns and recommendations

1. Statistical treatment and structure of the data

The authors base their analyses on 30 individual cells and apply one-way ANOVA followed by Dunnett’s multiple comparison test. While this approach is not inherently inappropriate for small datasets, its validity depends strongly on the underlying distributional assumptions and the independence and representativeness of the sampled observations.

A central issue is that different experimental conditions appear to produce fundamentally different data structures. Under control conditions (e.g., 0 Ca2+ or standard K+), GBFR values are relatively uniform across cells. In contrast, under high Ca2+ conditions, the response appears to split into two distinct populations, with cells showing either minimal exocytosis (GBFR near 100%) or a strong response (GBFR around 25%). This pattern is not compatible with a single homogeneous distribution and suggests condition-dependent heterogeneity in cellular states.

Under these circumstances, the use of mean-based ANOVA comparisons is not sufficient to describe the data. More importantly, summarizing such distributions into a single mean value risks obscuring biologically meaningful subpopulations. The authors should justify the statistical approach in light of these distributional differences and clarify whether assumptions of normality and homoscedasticity were formally tested. Alternative or complementary approaches that reflect the distributional structure should be considered.

(Response)

We thank the reviewer for this important comment. We agree that the original one-way ANOVA did not adequately account for the distributional heterogeneity observed among experimental conditions, particularly the large differences in variability among Ca2+ treatments. We have therefore substantially revised the statistical analyses. (Line 155-175)

First, multiple cells obtained from the same individual were not treated as independent biological replicates. We included individual identity as a random effect in all generalized linear mixed models (GLMMs), accounting for the non-independence of observations from the same individual and avoiding pseudoreplication.

Second, because the response variable represents the proportion of cells retaining or releasing globular bodies and substantial overdispersion was detected in the binomial models, the data were reanalyzed using beta-binomial GLMMs. For the Ca2+ experiments, residual diagnostics further indicated that the degree of dispersion differed among Ca2+ concentrations. We therefore fitted a beta-binomial GLMM in which the dispersion parameter was allowed to vary among Ca2+ treatments. Simulation-based residual diagnostics (DHARMa) indicated that this model adequately accounted for both the overall overdispersion and the treatment-dependent heterogeneity in residual variance.

The data shown in Figs. 2 and 5 were similarly reanalyzed using beta-binomial GLMMs when overdispersion was detected. Model-estimated proportions and 95% confidence intervals are now presented, and comparisons with the relevant control condition were performed using Dunnett-adjusted multiple comparisons.

We agree with the reviewer that the apparent bimodal responses observed under some conditions may reflect biological heterogeneity among cells. However, the present experiments do not allow us to determine whether this heterogeneity reflects discrete physiological states, differences in sperm maturity or condition, cellular damage, or other factors. We therefore did not assign a specific biological interpretation to the apparent bimodality. Instead, in the revised statistical analysis, we explicitly accounted for treatment-dependent heterogeneity in the response by allowing the dispersion parameter to vary among Ca2+ treatments in the beta-binomial GLMM. Our conclusions are therefore based on treatment-dependent differences in the estimated response rather than on the assumption that the observations represent distinct cellular subpopulations.

These revisions have also been reflected in the corresponding figures and figure legends. (Line 224-231; 293-301; 316-325; 343-353)

2. Effects of extracellular K+ and interpretation of variability

The effects of extracellular K+ are described mainly in terms of mean GBFR changes. However, the data indicate that K+ concentration also strongly affects the dispersion of responses.

At low K+ (artificial seawater), GBFR values are widely distributed (approximately 10–100%). With increasing K+ (20–100 mM), there is a progressive narrowing of this distribution, culminating in a highly constrained response range at high K+ (approximately 5–10% at 100 mM). This suggests not only a shift in average exocytotic activity but also a transition toward a more uniform cellular state.

This reduction in variability is not discussed in the manuscript, yet it may be as biologically relevant as the change in mean values. It suggests that extracellular K+ may act as a synchronizing factor for cellular response states rather than simply modulating exocytosis intensity.

In addition, the mechanistic interpretation attributing these effects to membrane potential changes remains speculative. No direct measurements of membrane potential or intracellular K+ levels are provided. This should be clearly acknowledged, and the interpretation should be adjusted accordingly.

(Response)

We appreciate the reviewer’s insightful comment. We agree that extracellular K+ affects not only the average globular body filling rate but also the apparent variability among observations. In the revised statistical analysis, we did not treat multiple sperm from the same individual as independent biological replicates; we included individual identity as a random effect in the beta-binomial generalized linear mixed model (GLMM). We used a beta-binomial model because the original binomial model showed overdispersion, and simulation-based residual diagnostics confirmed that the revised model adequately accounted for it.

The reduced variability observed at higher K+ concentrations is nevertheless biologically interesting. It may reflect reduced heterogeneity in cellular responses under these conditions. However, the present experiments do not allow us to determine whether this represents synchronization of physiological states, differences in sperm maturity or condition, or other sources of cellular heterogeneity. We have therefore added this possibility to the Discussion but have avoided interpreting the reduced variability as evidence for a specific physiological mechanism. (Line 460-463)

We also agree that our interpretation involving changes in membrane potential was too strong because we did not directly measure membrane potential and intracellular K+ in the present study. We have therefore revised the manuscript to describe this mechanism as a possibility rather than a demonstrated causal relationship. Direct measurements of membrane potential and ionic dynamics in parasperm will be an important subject for future investigation. (Line 456)

3. Role of Ca2+ and pharmacological evidence

The conclusion that Ca2+ plays a central role in globular body exocytosis is only partially supported by the presented data. While the absence of extracellular Ca2+ clearly abolishes exocytosis, graded Ca2+ effects are difficult to interpret due to heterogeneous and potentially bimodal responses at higher concentrations. Under these conditions, mean comparisons alone do not capture the structure of the data.

The pharmacological experiments using mibefradil and nifedipine are interpreted as evidence for involvement of T-type Ca2+ channels. However, this interpretation appears premature. Mibefradil is known to lack strict specificity at concentrations commonly used in physiological experiments and may also affect other channels, including Ca2+-activated K+ channels.

Given the strong sensitivity of the system to extracellular K+, an alternative explanation involving indirect modulation of K+-dependent processes cannot be excluded. This possibility is not addressed in the manuscript and should be discussed more explicitly.

Overall, the mechanistic conclusions regarding Ca2+ channel involvement would benefit from a more cautious formulation. Direct measurements of intracellular Ca2+ dynamics and intracellular K+ concentrations would be required to clearly distinguish between direct Ca2+ effects and indirect ionic interactions.

(Response)

We thank the reviewer for this important comment. We agree that the heterogeneous responses observed at higher CaCl2 concentrations cannot be adequately represented by mean comparisons alone. We therefore substantially revised the statistical analysis. The data were analyzed using a beta-binomial generalized linear mixed model (GLMM), with individual identity included as a random effect to account for multiple observations obtained from the same individual. Residual diagnostics indicated that the degree of dispersion differed among CaCl2 treatments; therefore, the beta-binomial dispersion parameter was allowed to vary among CaCl2 concentrations. Simulation-based residual diagnostics confirmed that this model adequately accounted for the treatment-dependent heterogeneity in the data. (Line 307-311; 329-335)

We also agree that the pharmacological results do not by themselves demonstrate a specific involvement of T-type Ca2+ channels. The effects of mibefradil and NNC55-0396 should be interpreted cautiously because pharmacological inhibitors may have off-target effects, and the present experiments cannot exclude indirect effects mediated through other ion-dependent processes, including K+-dependent mechanisms. We have therefore revised the Results and Discussion to avoid presenting T-type Ca2+ channel involvement as a demonstrated mechanism and instead describe the pharmacological results as being consistent with the possible involvement of these channels. (Line 441-445)

Similarly, because neither membrane potential nor intracellular Ca2+ or K+ dynamics were directly measured, we cannot distinguish direct Ca2+-dependent regulation from indirect interactions among ionic pathways. We have explicitly acknowledged this limitation in the revised Discussion and indicate that direct measurements of intracellular Ca2+, K+, and membrane potential will be required to resolve the underlying mechanism. (Line 463-464)

4. Motility analysis and missing quantitative parameters

The analysis of parasperm motility is limited to categorical descriptors (forward, backward, immotile). While informative at a basic level, this approach does not capture the full complexity of the observed responses.

Quantitative parameters such as swimming velocity, waveform propagation rate, or beat frequency would substantially strengthen the analysis. This is particularly important given the presence of heterogeneous cellular responses under certain conditions, where distinct functional subpopulations likely exist.

It would also be informative to examine potential correlations between motility patterns, GBFR values, and response heterogeneity. Such an integrated analysis could help determine whether exocytosis state and motility behavior are mechanistically linked or represent independent response pathways.

The current data suggest that high extracellular K+ induces a relatively uniform forward motility state, whereas mibefradil produces a mixed phenotype with immotile and motile subpopulations. Ca2+-free conditions largely abolish motility. These observations raise additional questions regarding the specificity of pharmacological effects and should be discussed more cautiously.

(Response)

The swimming velocity and waveform propagation rate have been described in our previous paper (Shibata, 2022). In response to the reviewer’s comment, we have now presented the motility data side by side across the different experimental series to facilitate comparison. We have also measured motility (swimming velocity) and added the results as a table, with the data presented as mean ± SD.

It is an interesting question whether the state of exocytosis and swimming behavior are mechanistically linked or instead represent independent response pathways. Although these two processes may involve somewhat different signaling mechanisms, their temporal dynamics also differ. We have therefore added a discussion of this point in the revised manuscript. (Line 472-478)

As the reviewer points out, mibefradil induces a mixed phenotype consisting of both non-motile and motile subpopulations. Under Ca2+-free conditions, motility was almost completely abolished. This may suggest some degree of toxicity under these conditions, although it is also possible that sperm from the vas deferens exist in different physiological states. The variability in motility observed under ASW conditions may support the latter possibility. At present, however, it is difficult to investigate these possibilities in greater depth, and we have therefore limited our discussion of these issues to the Discussion section. (Line 460-464)

Revised closing and recommendations (to replace final paragraphs)

Overall, this manuscript addresses an interesting and potentially important aspect of gastropod reproductive biology. The experimental system is valuable, and the dataset contains observations that could meaningfully advance understanding of parasperm function and ion-dependent regulation of exocytosis and motility.

However, in its current form, the main limitation is not the experimental system itself, but the level of analysis and interpretation. The results indicate clear heterogeneity in cellular responses and strong ion-dependent effects, but these patterns are not sufficiently quantified, integrated, or mechanistically resolved. As a result, several of the central conclusions remain speculative or are not fully supported by the presented data.

For the manuscript to reach the level required for publication, the following issues need to be addressed:

• The statistical analysis must be revised to properly reflect the structure and distribution of the data, including explicit consideration of non-uniform and potentially bimodal response patterns.

(Response)

As described in detail above, we substantially revised the statistical analyses to account for the non-independence and heterogeneous distribution of the observations. We did not treat multiple sperm from the same individual as independent biological replicates; we included individual identity as a random effect in the generalized linear mixed models (GLMMs). Because we detected substantial overdispersion in the binomial models, we analyzed the data using beta-binomial GLMMs.

For the experiments examining Ca2+ dependence, residual diagnostics further indicated that dispersion differed among Ca2+ concentrations. We therefore allowed the beta-binomial dispersion parameter to vary among Ca2+ treatments. Simulation-based residual diagnostics confirmed that this model adequately accounted for the treatment-dependent heterogeneity in the data.

We acknowledge the reviewer’s observation that the distributions under some conditions appear non-uniform and potentially bimodal. However, the present data do not allow us to determine whether this pattern represents discrete physiological subpopulations or other sources of cellular heterogeneity. We therefore do not interpret the apparent distributional pattern as evidence for distinct cellular populations. Rather, our revised statistical analysis accounts for the observed extra-binomial and treatment-dependent variability, and our conclusions are restricted to treatment-dependent differences supported by these analyses.

• The interpretation of K+-dependent effects requires direct physiological validation. At minimum, intracellular K+ levels or membrane potential changes should be measured under key conditions, including high K+ treatment and mibefradil exposure.

• Ca2+-dependent conclusions should be supported by direct measurements of intracellular Ca2+ dynamics across the relevant experimental conditions, rather than inferred solely from extracellular manipulation.

(Response)

We have attempted to perform imaging experiments to monitor these ionic dynamics but encountered several technical difficulties. As the reviewer points out, such experiments would be very important for directly addressing the underlying mechanisms. However, we would like to leave these experiments as an important subject for future investigation. We have added a discussion of this issue to the revised manuscript. (Line 463-464)

• Motility analysis should be extended beyond categorical descriptors to include quantitative parameters (e.g., velocity and/or waveform propagation metrics), allowing proper comparison between functional states.

(Response)

The swimming velocity and waveform propagation rate have been described in our previous paper (Shibata, 2022). In response to the reviewer’s comment, we have now presented the motility data side by side across the different experimental series to facilitate comparison. We have also measured motility (swimming velocity) and added the results as a table. (Table 1; Line 365-374)

• The apparent presence of distinct cellular subpopulations should be explicitly characterized rather than treated implicitly through mean-based comparisons. Integration of exocytosis and motility data is particularly important to determine whether these represent functionally distinct physiological states.

(Response)

We thank the reviewer for this important and insightful suggestion. We carefully considered how best to integrate the exocytosis and motility data and, as a result, have added a summary of these data as a table in Figure 9A. We believe that this presentation also makes the relationship between these experimental findings and the proposed model in Figure 9B clearer.

After these points are addressed and the datasets are integrated in a more quantitative and mechanistically grounded way will it be possible to properly define the biological role of Ca2+ and K+ in regulating parasperm function. With these revisions, the manuscript has strong potential to become a solid and valuable contribution to the field.

Attachment Submitted filename: Response_to_Reviewers.docx

# Peer review: Decision Letter 1

**DOI:** 10.1371/journal.pone.0360627.r003

**Authors:** Sayyed Mohammad Hadi Alavi

29 Sep 2026

Ca2+-dependent exocytosis of parasperm globular bodies in the strawberry conch gastropod Conomurex luhuanus

PONE-D-26-23225R1

Dear Dr. Inaba,

We’re pleased to inform you that your manuscript has been judged scientifically suitable for publication and will be formally accepted for publication once it meets all outstanding technical requirements.

Within one week, you’ll receive an e-mail detailing the required amendments. When these have been addressed, you’ll receive a formal acceptance letter and your manuscript will be scheduled for publication.

An invoice will be generated when your article is formally accepted. Please note, if your institution has a publishing partnership with PLOS and your article meets the relevant criteria, all or part of your publication costs will be covered. Please make sure your user information is up-to-date by logging into Editorial Manager at Editorial Manager® and clicking the ‘Update My Information' link at the top of the page. For questions related to billing, please contact billing support.

If your institution or institutions have a press office, please notify them about your upcoming paper to help maximize its impact. If they’ll be preparing press materials, please inform our press team as soon as possible -- no later than 48 hours after receiving the formal acceptance. Your manuscript will remain under strict press embargo until 2 pm Eastern Time on the date of publication. For more information, please contact onepress@plos.org.

Kind regards,

Sayyed Mohammad Hadi Alavi, PhD

Academic Editor

PLOS One

Additional Editor Comments (optional):

Authors have addressed all comments raised by the reviewers and the manuscript now looks suitable for publication.

Reviewers' comments:

Reviewer's Responses to Questions

**Comments to the Author**

Reviewer #1: All comments have been addressed

Reviewer #2: All comments have been addressed

**********

2. Is the manuscript technically sound, and do the data support the conclusions?

Reviewer #1: Yes

Reviewer #2: Yes

**********

3. Has the statistical analysis been performed appropriately and rigorously?

Reviewer #1: Yes

Reviewer #2: Yes

**********

4. Have the authors made all data underlying the findings in their manuscript fully available?

The PLOS Data policy

Reviewer #1: Yes

Reviewer #2: Yes

**********

5. Is the manuscript presented in an intelligible fashion and written in standard English?

Reviewer #1: Yes

Reviewer #2: Yes

**********

Reviewer #1: The authors have carefully addressed my major concerns and substantially improved the manuscript. The manuscript is substantially improved and is suitable for publication in its current form.

Reviewer #2: The revised manuscript has been substantially improved and addresses my major concerns. I have no further concerns, and I consider the manuscript acceptable for publication.

**********

what does this mean?). If published, this will include your full peer review and any attached files.

If you choose “no”, your identity will remain anonymous but your review may still be made public.

**Do you want your identity to be public for this peer review?**  For information about this choice, including consent withdrawal, please see our Privacy Policy

Reviewer #1: No

Reviewer #2: No

**********

# Editor report: Acceptance letter

**DOI:** 10.1371/journal.pone.0360627.r004

**Authors:** Sayyed Mohammad Hadi Alavi

PONE-D-26-23225R1

PLOS One

Dear Dr. Inaba,

I'm pleased to inform you that your manuscript has been deemed suitable for publication in PLOS One. Congratulations! Your manuscript is now being handed over to our production team.

At this stage, our production department will prepare your paper for publication. This includes ensuring the following:

* All references, tables, and figures are properly cited

* All relevant supporting information is included in the manuscript submission,

* There are no issues that prevent the paper from being properly typeset

You will receive further instructions from the production team, including instructions on how to review your proof when it is ready. Please keep in mind that we are working through a large volume of accepted articles, so please give us a few days to review your paper and let you know the next and final steps.

Lastly, if your institution or institutions have a press office, please let them know about your upcoming paper now to help maximize its impact. If they'll be preparing press materials, please inform our press team within the next 48 hours. Your manuscript will remain under strict press embargo until 2 pm Eastern Time on the date of publication. For more information, please contact onepress@plos.org.

You will receive an invoice from PLOS for your publication fee after your manuscript has reached the completed accept phase. If you receive an email requesting payment before acceptance or for any other service, this may be a phishing scheme. Learn how to identify phishing emails and protect your accounts at https://explore.plos.org/phishing.

If we can help with anything else, please email us at customercare@plos.org.

Thank you for submitting your work to PLOS One and supporting open access.

Kind regards,

PLOS One Editorial Office Staff

on behalf of

Dr. Sayyed Mohammad Hadi Alavi

Academic Editor

PLOS One
